# Manufacturing CD20/CD19-targeted iCasp9 regulatable CAR-T_SCM_ cells using a *Quantum pBac*-based CAR-T engineering system

**DOI:** 10.1371/journal.pone.0309245

**Published:** 2024-08-27

**Authors:** Peter S. Chang, Yi-Chun Chen, Wei-Kai Hua, Jeff C. Hsu, Jui-Cheng Tsai, Yi-Wun Huang, Yi-Hsin Kao, Pei-Hua Wu, Po-Nan Wang, Yi-Fang Chang, Ming-Chih Chang, Yu-Cheng Chang, Shiou-Ling Jian, Jiann-Shiun Lai, Ming-Tain Lai, Wei-Cheng Yang, Chia-Ning Shen, Kuo-Lan Karen Wen, Sareina Chiung-Yuan Wu

**Affiliations:** 1 GenomeFrontier Therapeutics TW Co., Ltd., Taipei City, Taiwan (R.O.C.); 2 Division of Hematology, Chang Gung Medical Foundation, Linkou Branch, Taipei City, Taiwan (R.O.C.); 3 Division of Hematology and Oncology, Department of Internal Medicine, Mackay Memorial Hospital, Taipei, Taiwan (R.O.C.); 4 Department of Medical Research, Laboratory of Good Clinical Research Center, Mackay Memorial Hospital, Tamsui District, New Taipei City, Taiwan (R.O.C.); 5 Department of Medicine, Mackay Medical College, New Taipei City, Taiwan (R.O.C.); 6 OBI Pharma, Inc., Taipei, Taiwan (R.O.C.); 7 Biomedical Translation Research Center, Academia Sinica, Taipei, Taiwan (R.O.C.); 8 Genomics Research Center, Academia Sinica, Taipei, Taiwan (R.O.C.); University of California Santa Barbara, UNITED STATES OF AMERICA

## Abstract

CD19-targeted chimeric antigen receptor (CAR) T cell therapies have driven a paradigm shift in the treatment of relapsed/refractory B-cell malignancies. However, >50% of CD19-CAR-T-treated patients experience progressive disease mainly due to antigen escape and low persistence. Clinical prognosis is heavily influenced by CAR-T cell function and systemic cytokine toxicities. Furthermore, it remains a challenge to efficiently, cost-effectively, and consistently manufacture clinically relevant numbers of virally engineered CAR-T cells. Using a highly efficient *piggyBac* transposon-based vector, *Quantum pBac*™ (*qPB*), we developed a virus-free cell-engineering system for development and production of multiplex CAR-T therapies. Here, we demonstrate *in vitro* and *in vivo* that consistent, robust and functional CD20/CD19 dual-targeted CAR-T stem cell memory (CAR-T_SCM_) cells can be efficiently produced for clinical application using *qPB*™. In particular, we showed that *qPB*™-manufactured CAR-T cells from cancer patients expanded efficiently, rapidly eradicated tumors, and can be safely controlled via an iCasp9 suicide gene-inducing drug. Therefore, the simplicity of manufacturing multiplex CAR-T cells using the *qPB*™ system has the potential to improve efficacy and broaden the accessibility of CAR-T therapies.

## Introduction

Over the last decade, chimeric antigen receptor (CAR)-T therapy has become one of the most promising cancer treatments, but the success achieved by CAR-T therapy in B-cell lymphomas has not been replicated in solid tumors [1–3]. Moreover, the heavy reliance on lentiviral and retroviral vectors for CAR-T cell manufacturing makes the therapy expensive and inaccessible to many patients [4]. Frequent cytokine release syndrome (CRS) and immune effector cell-associated neurotoxicity syndrome (ICANS) are associated with conventional CAR-T therapies, raising important safety concerns [5]. Furthermore, virus-based gene therapies have limited gene payload capacity, making them less suitable for engineering multiplex armored CAR-T cells [6, 7].

Transposon systems, such as *Sleeping Beauty* and *piggyBac*, are virus-free alternatives for generating CAR-T cells, but these systems often have limitations such as low gene transfer efficiency and limited expansion of engineered cells [6, 8, 9]. While the manufacture of clinically relevant numbers of high-quality CAR-T cells from senescent and exhausted patient T cells is challenging with virus-based cell-engineering, the challenge is even greater when using many virus-free methods. The poor gene delivery rates and severe cell damage associated with electroporation of virus-free vectors typically lead to low CAR-T yield. One approach to expanding the CAR^+^ T cell population is by culturing the cells with artificial antigen presenting cells (aAPC), but this method often leads to a more differentiated and short-lived T_EFF_/exhausted phenotypes [10]. Another approach is to include markers within the CAR-T construct for drug selection or antibody-mediated cell isolation during or after CAR-T *ex vivo* expansion. However, such approaches increase the payload size and introduce therapeutically irrelevant genes into the genome [7, 11, 12].

To overcome the limitations of transposon systems, we recently developed an advanced version of the *piggyBac* system known as *Quantum pBac*™ (*qPB*). This binary *piggy-Bac* system comprises a minicircle donor vector and a helper plasmid expressing a molecular-engineered *piggyBac* transposase, *Quantum PBase*™ (*qPBase*) [13, 14]. Two versions of this transposase, *qPBase v1 and qPBase* v2, were respectively derived from wild-type piggyBac and hyperactive transposase (*hyPBase*) via fusion with GFP [14]. We further showed that *qPBase v2* mediates significantly higher transposition activities compared to conventional hyperactive *piggyBac* transposase (*hyPBase*) in CAR-T cell production [13]. Furthermore, our previous work showed that *qPB-*modified CAR-T cells predominantly exhibit an efficacious T_SCM_ phenotype and can eradicate tumors both *in vitro* and *in vivo*. T_SCM_ is a class of immunological memory T cells, and enrichment of this class can improve the success of adoptive T cell therapies [15–19]. However, we also observed donor-dependent variability in the percentage of CAR-T_SCM_ cells among total CAR^+^ cells and in the performance of *qPB-*modified CAR-T cells. These drawbacks have potential to severely hamper clinical translation of this technology.

For development of CAR-T cell technologies, B-cell malignancies are among the most actively studied cancers. Since Brentjens et al. first demonstrated the eradication of B-cell tumors by CD19-targeted second-generation CAR-T cells, several commercial CAR-T products have been approved by regulatory agencies [20, 21]. However, treatment with CD19-directed CAR-T therapy currently results in only 30–40% long-term progression-free survival in aggressive Non-Hodgkin lymphoma (NHL) patients, and the median event-free survival of adult B-cell ALL patients is only 6.1 months [22, 23]. Evidence suggests that downregulation of CD19 may be in part responsible for patient relapse [24, 25]. In line with this idea, recent clinical studies have shown that CD19 and CD20 dual-targeting CAR-T therapy can improve patient survival compared to CD19-directed CAR-T therapy [26, 27].

In this report, we detail further advancements in our *qPB*-based system using a multiplex gene of interest that incorporates iCasp9 suicide gene and CD19/CD20 dual targeting CARs for treatment of B-cell malignancy. We first show that *qPB*-modified CAR-T manufacturing can be improved with *Quantum Booster™* (*qBT*), a supplement containing nutrients that promote robust CAR-T expansion and enrich the CAR^+^ T_SCM_ population. We further observed enhanced anti-tumor efficacy of *qPB*-modified CAR-T cells in both *in vitro* and *in vivo* xenotransplant immunodeficient mouse models of B-cell malignancy. The superior quality of *qPB*-modified CAR-T cells produced with *qBT* was confirmed in a solid tumor xenograft model. Additionally, we found that clinically relevant numbers of highly potent CAR-T_SCM_ cells could be rapidly generated from cancer patients using G-Rex culture vessels. By shortening the developmental timeline of virus-free multiplex CAR-T therapy, the *qPB* system represents a technological advancement for CAR-T generation with the potential to facilitate widespread patient access.

## Materials and methods

### Generation/expansion of CAR-T cells

Human blood samples were collected in January-April 2020 and March-April 2021. Frozen peripheral blood mononuclear cells (PBMCs) from adult healthy donors/patients were obtained from Chang Gung Memorial Hospital (IRB#: 201900578A3) and MacKay Memorial Hospital (IRB#: 20MMHIS330e). PBMCs were extracted using Ficoll-Hypaque gradient separation (Cytiva). Primary human CD3^+^ T cells were isolated from PBMCs by negative selection (EasySep Human T Cell Isolation Kit, StemCell Technologies) or by positive selection (Dynabeads Human T-Expander CD3/CD28, Thermo Fisher), and activated with Dynabeads (3:1 bead to cell ratio). An iCasp9-CD20/CD19-CAR transposon minicircle carrying CD20 scFv[1] and CD19 scFv[2] and a *qPBase* plasmid were delivered into activated T cells using 4D-Nucleofector device (Lonza) and *Quantum Nufect*™ (GenomeFrontier). Irradiated CD19-expressing K562 cells (aAPC) were added to electroporated T cells (1:1) on day 3. Cells were cultured in OpTmizer medium (Thermo) supplemented with *Quantum Booster* (GenomeFrontier) under fed-batch or perfusion culture condition for 10 days. For lentiviral CAR-T production, primary human CD3^+^ T cells isolated from cryopreserved human PBMCs (human Pan T Cell Isolation Kit, Miltenyi Biotec, 130-096-535) were activated and expanded with Dynabeads (1:1), and transduced with 1 MOI concentrated virus containing 8 μg/mL Polybrene (Merck, TR-1003-G). CAR-T cells were then cultured in IL-7- and IL-15-supplemented media until harvest/use.

### Cell culture conditions

#### Fed-batch

On day 1, 1 × 10^5^ nucleofected cells were seeded into a well of 24-well G-Rex (Wilson Wolf) containing 3 ml of medium. Fresh medium (2 ml) was added on days 3 and 5, followed by replacement of 2 ml medium on day 7.

#### Perfusion

On day 1, nucleofected cells were seeded into conventional 24-well plates at a cell concentration of 2.5 × 10^5^ cells/ml. Every 2–3 days, the cell numbers were counted, and fresh medium was added to maintain cell concentration at 2.5 × 10^5^ cells/ml. Then, cells were placed back into culture; depending on the volume, a culture dish of up to 100-mm diameter was used [28, 29]. For both of the culture strategies, cells were harvested on day 10 for use in subsequent experiments.

### Flow cytometry

CAR expression was assessed as described previously [13]. Briefly, CAR expression on T cells was detected by flow cytometry following staining of cells at 4°C for 30 min with F(ab’)2 fragment-specific, biotin-conjugated goat anti-mouse antibodies (Jackson ImmunoResearch Laboratories) and R-phycoerythrin (PE)-conjugated streptavidin (Jackson ImmunoResearch Laboratories). Surface molecule expression was determined by staining T cells with fluorochrome-conjugated antibodies. The following antibodies were used: CD3-PE-Cy5, CD4-AlexaFluor532, CD4-AlexaFlour700, CD8-BV605, CD8-Pacific Blue, CD27-PE-Cy7, CD28-APC, CD45RO-PE, CD62L-PE-Dazzle594, CD197-BV510, CD279/PD-1-PE/Cy7, CD366/Tim-3-BV650, CD223/LAG-3-BV711, KLRG-1-BV-421, CD57-PerCP-Cy5.5, CD19-PE, CD56-BV711 (BioLegend), CD45RA-BUV737, and CD95-BUV395 (BD Biosciences). Ghost dye Red780 fixable viability dye (Cytek), 7AAD or propidium iodide (PI) was used to determine cell viability. Mouse blood cell samples were stained with anti-hCD45-APC antibody (BioLegend). Flow cytometry data were acquired on an SA3800 Spectral Analyzer (Sony), a BD FACSCantoTM II (BD), or a BD LSRFortessa analyzer (Sony).

### Enzyme-linked immunosorbent assay (ELISA)

For the *in vitro* assay, supernatant was collected after 24 hours of CAR-T and Raji-GFP/Luc cell (Creative Biogene, CSC-RR0320) co-culture (E:T = 1:1). For the *in vivo* assay, mouse plasma samples were collected on days 2, 5, 8, and 14 after T cell injection. IFN-γ (Thermo), TNF-α (Thermo), IL-6 (Thermo), and IL-2 (BioLegend) were quantified by ELISA according to the manufacturer’s instructions.

### Genomic DNA extraction and quantitative PCR (qPCR)

Genomic DNA from mouse blood was extracted using DNeasy Blood & Tissue Kit (Qiagen). For qPCR analysis, CAR-T cells and Raji-GFP/Luc cells were assessed using the following primer sequences: CAR: fwd-5’-ACGTCGTACTCTTCCCGTCT-3’, rev-5’-GATCACCCTGTACTGCAACCA-3’; Luciferase: fwd-5’-GGACTTGGACACCGGTAAGA-3’, rev-5’-GGTCCACGATGAAGAAGTGC-3’. qPCR assays were performed using a 7500 fast real-time PCR system (Applied Biosystems). Absolute transgene levels were calculated using the standard curve method. The threshold cycle (C_T_) value of each target was converted to a concentration using the appropriate standard curve. The percentage of cells expressing CAR was calculated according to copy number per cell.

### *In vitro* cytotoxicity assay

Cytotoxicity was assessed using Celigo image cytometry (Nexcelom) as previously described [13]. CAR-T cells were added to Raji-GFP/Luc cells at 5:1, 1:1, and 1:5 E:T ratios.

### Anti-tumor efficacy in mouse tumor models

**Fig 1:**
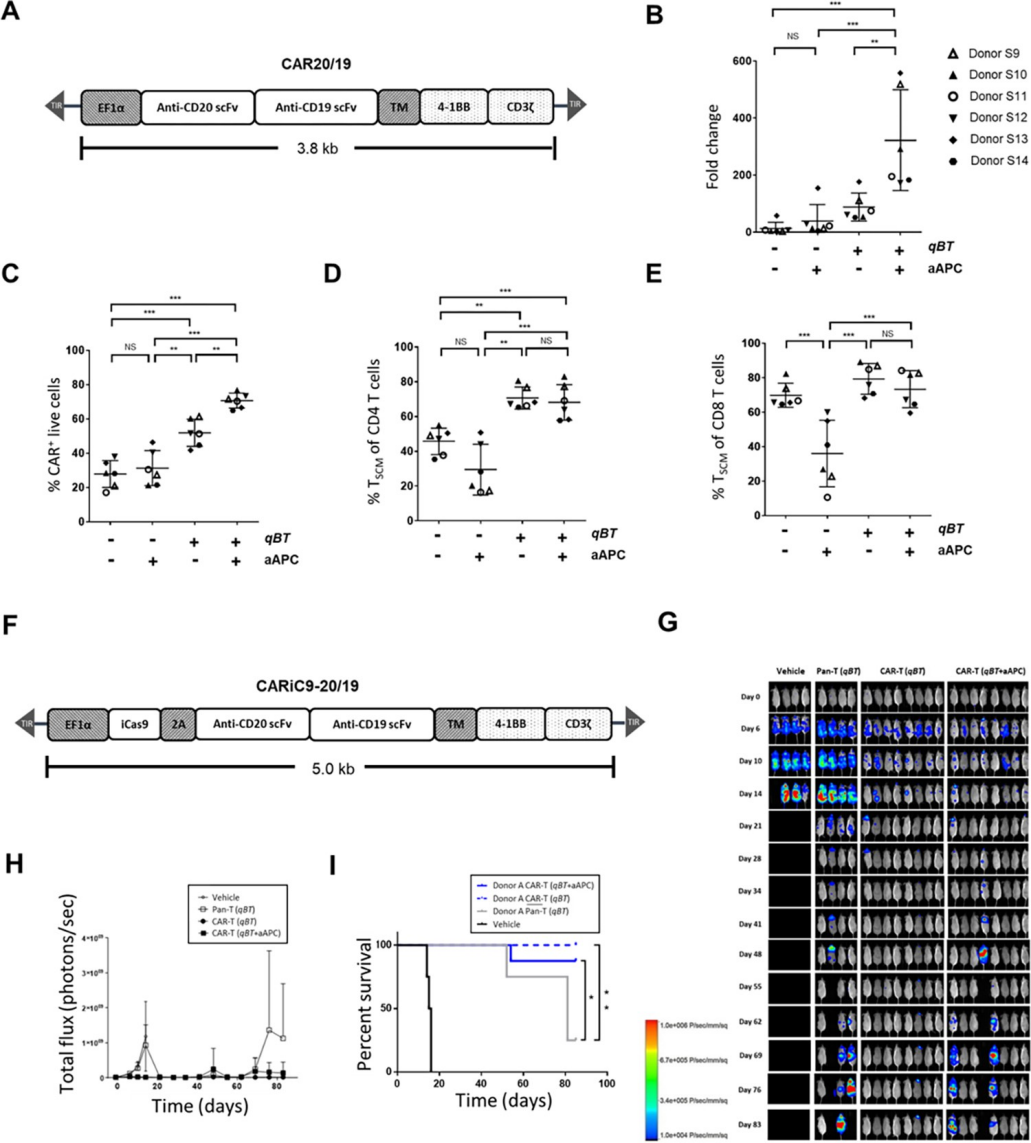
Effect of *qBT* and aAPC on CAR-T cells. (A) Schematic diagram of the CAR20/19 CAR construct. (B-E) Human peripheral blood mononuclear cells (PBMC) electroporated with *Quantum pBac*™-expressing CAR and cultured for 10 days in the presence or absence of aAPC and/or *Quantum Booster*™ (*qBT*) were harvested and assessed for (B) cell expansion fold change, (C) percentage CAR^+^ of live cells (CD3^+^ PI^-^, >90%), and percentage of T_SCM_ cell subsets in (D) CD4^+^ and (E) CD8^+^ cells. Data shown are from six healthy donors. Horizontal lines represent the mean and s.e.m. fold change (B), mean and s.e.m. percentage of CAR^+^ cells (C), and mean and s.e.m. percentage of T_SCM_ cell subsets (D and E). * *p <* 0.05, ** *p <* 0.01, *** *p <* 0.001. (F) Schematic diagram of the CARiC9-20/19 CAR construct. A T2A sequence enables co-expression of iCasp9 and CD20/CD19-targeted scFvs under the EF1α promoter. (G-I) *In vivo* functional characterization of CAR-T cells produced by perfusion culture system. (G) *In vivo* cytotoxicity of CAR-T cells with or without pre-incubation with aAPC in Raji-GFP/Luc-bearing immunodeficient mice. Fluorescence intensity values (H) and survival curves (I) of mice from (G) plotted against time. Results shown are from 4 to 8 mice/group. T cells were obtained from a representative donor. Vehicle and Pan-T cells (non-engineered T cells) were used as controls. *qBT* was present in all cell culture conditions. Groups were compared by log-rank (Mantel-Cox) test, *p < 0.05, **p < 0.01.

Studies on *in vivo* mouse xenograft models were conducted as previously described [13] at the Development Center for Biotechnology (Taiwan) using Taiwan Mouse Clinic IACUC (2020-R501-035) approved protocols. Six- to eight-week-old male immunodeficient (NOD.Cg-Prkdc^scid^Il2rg^tm1Wjl^/YckNarl) mice (National Laboratory Animal Center, Taiwan) were engrafted by intravenous (*i*.*v*.) injection of 1.5 × 10^5^ Raji-GFP/Luc tumor cells or by subcutaneous (*s*.*c*.) injection of Matrigel (1:1, BD Bioscience) containing 2 × 10^6^ NCI-N87-Luc cells on the right flank. For the Raji tumor model in **Fig 1**, mice were infused one week after engraftment with 3 × 10^6^ CAR-T cells or control Pan-T cells (non-transfected T cells). The duration of the experiment was 90 days. Twenty-four mice were used in the experiment; all 24 mice survived the study and were euthanized at the conclusion of the experiment. For the Raji tumor model in **Fig 4**, mice were infused one week after engraftment with 7.5 × 10^5^ (low dose), 3 × 10^6^ (medium dose), or 1 × 10^7^ (high dose) CAR-T cells. After 35 days, 1.5 × 10^5^ Raji-GFP/Luc tumor cells were injected (*i*.*v*.) to half of the mice in each group. The duration of the experiment was 98 days. Twenty-eight mice were used in the experiment; 28 mice were euthanized, and none were found dead. Raji-GFP/Luc tumor cells were quantified using the Xenogen-IVIS Imaging System (Caliper Life Sciences). For the NCI-N87 tumor model in **Fig 5**, mice were injected *i*.*v*. 11 days after engraftment with 4 × 10^6^ control or CAR-T cells. The duration of the experiment was 48 days. Fifteen mice were used in the experiment; 15 mice were euthanized, and none were found dead. Mice were imaged on an Ami-HT optical imaging system (Spectral Instruments Imaging), following intraperitoneal (*i*.*p*.) injection of D-Luciferin (BIOSYNTH, L-8220). In all mouse studies, animal health and behavior were monitored daily. A warm pad was provided, and animal feed was replaced with nutritionally fortified water gel when animals were observed to be weak/lethargic. An animal was euthanized if one of the following conditions occurred: 1) the appearance of abnormal movement or paralysis, or 2) body weight loss exceeded 20% of the weight at initial treatment. Animals were euthanized within one day after reaching endpoint criteria.

### *In vitro* iCasp9 safety assay

Activation of inducible caspase 9 (iCasp9) was performed *in vitro* with 2.5, 5, or 10 nM of dimerizer (AP1903), and CAR-T cell elimination was assessed by flow cytometry 24 hours later.

### ddPCR copy number assays

Following genomic DNA (gDNA) extraction from CARiC9-20/19 CAR-T cells, minicircle DNA (positive control) was manufactured by Aldevron. Before ddPCR, minicircle DNA was digested for 1 min at 37°C with BsrGI (Thermo). Each reaction for ddPCR Copy Number Variation Assay was set up in 20 μl sample volume containing 10 μl of 2X ddPCR Supermix (no dUTP, BioRad), 1 μl of FAM-labeled target primers/probe (BioRad), 1 μl of HEX-labeled reference primers/probe (BioRad), 0.1 μl of BsrGI, and 50 ng of gDNA or minicircle DNA (volume 2 μl). *RPP30* was used as a housekeeping gene. BsrGI completes restriction digests within the ddPCR reaction mixture. Droplets were generated in a QX200 droplet generator (BioRad). After DNA amplification, droplets were analyzed in a QX200 droplet reader (BioRad). Data analysis was performed with QuantaSoft (BioRad).

### Statistical analysis

Statistical analysis was performed using GraphPad Prism 7. Two-tailed unpaired t-tests were used to compare between two groups. One-way ANOVA was used for comparing three or more groups in a single condition. *P* ≤ 0.05 was considered statistically significant; significance is indicated as *p < 0.05, **p < 0.01, and ***p < 0.001. The data generated for this study will be made available by the corresponding author upon reasonable request.

## Results

### *Quantum Booster™* (*qBT*) enriches the percentage of CAR^+^ T_SCM_ and enhances anti-tumor efficacy of CAR-T cells

We previously demonstrated that *qPB* may be used for clinical application in CAR-T therapy [13]. However, even in the presence of artificial antigen presenting cells (aAPC), T cells from some donors still failed to sufficiently expand. To address the difficulty of manufacturing sufficient quantities of high-quality CAR-T cells for clinical application, we developed a serum-free CAR-T cell culture supplement named *Quantum Booster™ (qBT)*, which contains cellular proteins and a set of cytokines at defined concentrations. The components of *qBT* promote proliferation of the CAR-T cells while maintaining stemness, limiting senescence and preventing exhaustion. We analyzed the effects of *qBT*, aAPC, and the combination (aAPC+*qBT*) on characteristics and performance of *qPB*-produced CAR-T cells engineered with CAR20/19 DNA, a second-generation bicistronic CAR containing binding domains that recognize CD20 and CD19 driven by the EF1α promoter (**Fig 1A**). The results showed that *qBT* increased the production and expansion of CAR^+^ T cells (**Fig 1B and 1C**). While culture with aAPC alone did not significantly improve production and expansion of CAR^+^ T cells, aAPC+*qBT* significantly enhanced both of these parameters. More importantly, *qBT*-cultured CAR-T cells were characterized by high percentages of CD45RA^+^CD62L^+^CD95^+^ T_SCM_ cells in both the CD4^+^ and CD8^+^ populations (**Fig 1D and 1E**). In contrast, cells cultured with aAPC alone had markedly lower percentages of T_SCM_ cells, suggesting that aAPC may promote T cell maturation. Furthermore, cells cultured with aAPC+*qBT* did not show reduced percentages of T_SCM_ cells, suggesting that *qBT* maintains cells in a less differentiated T_SCM_ state (**Fig 1D and 1E**). These results demonstrated that *qBT* markedly enhances the expansion capacity of CAR-T cells and preserves CAR-T quality.

We next compared the anti-tumor activities of CAR-T cells cultured with *qBT* alone and those cultured with *qBT* in combination with aAPC (*qBT*+aAPC) using an *in vivo* xenotransplant Raji tumor model. For this purpose, we engineered CAR-T cells with CARiC9-20/19, a second-generation bicistronic CAR containing binding domains that recognize CD20 and CD19 as well as an iCasp9 suicide gene driven by the EF1α promoter **(Fig 1F)**. Xenografted mice were treated with vehicle, Pan-T, or CAR-T cells cultured with *qBT* or *qBT*+aAPC. The results confirmed that CAR-T cells cultured in either *qBT* alone or *qBT* in combination with aAPC (aAPC+*qBT*) both exhibited enhanced anti-tumor efficacy and prolonged survival of treated mice compared to the control (**Fig 1G–1I**). Notably, there was a slightly greater anti-tumor efficacy seen in *qBT* cultured CAR-T cells than in *qBT*+aAPC cultured CAR-T cells. Given that *qBT* markedly enhanced the expansion and quality of CAR-T cells, all subsequent experiments were conducted using *qPB*-engineered CAR-T cells cultured with *qBT*.

### Comparison of fed-batch (G-Rex) and perfusion (conventional)-cultured CAR-T cells

Maintaining sterility and controlling mycoplasma and endotoxin contamination in the CAR-T product are of utmost importance for safe manufacturing. G-Rex is a good manufacturing practice (GMP)-compliant cell culture vessel used for cell expansion [28, 29]. The minimal manual operation of this culture vessel reduces contamination risks compared to perfusion-cultured flasks. Using CARiC9-20/19, we assessed *qPB*-engineered CAR-T cells expanded in G-Rex (fed-batch) and conventional (perfusion) plates (**S1A Fig**). We observed comparable percentages of CAR^+^ and T_SCM_ cells with greater cytotoxicity among the G-Rex CAR-T cells (**S1B Fig**). As G-Rex will be adopted for GMP CAR-T manufacturing, all subsequent experiments were conducted using cells expanded with G-Rex.

### Safety assessment of CARiC9-20/19 CAR-T cells

As a means to better manage CAR-T therapy-associated adverse events, the iCasp9 suicide gene was included in the CARiC9-20/19 construct. We demonstrated that AP1903 treatment, which activates iCasp9, eradicated CAR-T cells (**S2A Fig**). To comply with GMP safety standards for CAR-T manufacturing, we modified CAR-T cells using different amounts of CARiC9-20/19 DNA and determined that the CAR copy number remained low (<5 copies per cell) when 15 μg of total DNA was used for nucleofection (**S2B Fig**). All subsequent experiments were conducted using 15 μg of DNA per 5 × 10^6^ cells in order to maximize CAR^+^ T cell production while maintaining safe levels of CAR DNA copy numbers.

### CARiC9-20/19 CAR-T cells are dominated by T_SCM_ cells with high proliferative capacity, low exhaustion and senescence, and enhanced cytotoxic function

**Fig 2:**
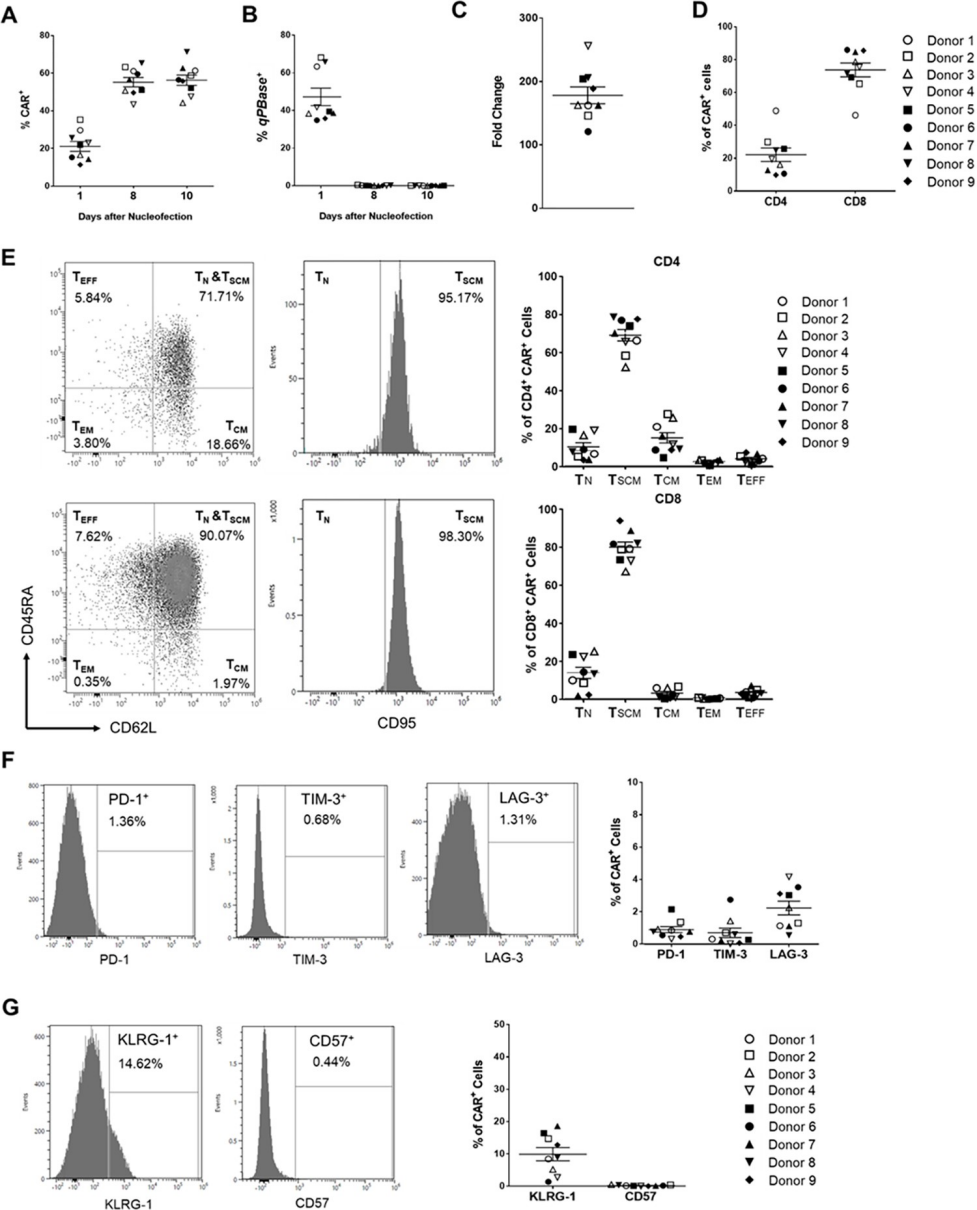
Characteristics of CARiC9-20/19 CAR-T cells. Percentages of (A) CAR^+^ and (B) transposase^+^ (*qPBase*^+^) cells were analyzed by flow cytometry on days 1, 8 and 10 after nucleofection. (C) Fold change of CARiC9-20/19 CAR-T cells after 10 days of culture in G-Rex are shown. (D) Percentage of CD4 and CD8 T cells in the CAR^+^ population on day 10 after nucleofection. (E) Distributions of T_N_, T_SCM_, T_CM_, T_EM_, and T_EFF_ subsets in the CD4 (upper panel) and CD8 populations (lower panel). (F) Expression of exhaustion markers PD-1, TIM-3, and LAG-3 in CARiC9-20/19 CAR-T cells at 10 days post-nucleofection. (G) Expression of senescence markers KLRG-1 and CD57 in CARiC9-20/19 CAR-T cells. (A-G) Data represent mean ± SD for 9 healthy donors, n = 9. Each set of histogram plots shown in (C), (D), (E), and (F) represents one donor.

We next examined the performance of healthy donor-derived CAR-T cells. We observed 21.49% (±7.87%, n = 9) CAR^+^ T cell population on day 1, which subsequently increased and stabilized at 55.7% (±7.33%, n = 9) and 56.75% (±8.20%, n = 9) on days 8 and 10, respectively (**Fig 2A**). As the long-term presence of transposase (*Quantum PBase*, or *qPBase*) in modified T cells may lead to genotoxicity, we also monitored the *qPBase* levels following nucleofection. While the *qPBase* levels were high on day 1 (47.31% ± 14.02%, n = 9), insignificant levels (<0.2%) of *qPBase*^+^ cells remained on days 8 and 10, suggesting there is minimal concern regarding *qPBase*-induced integrant remobilization (**Fig 2B**). We also observed an average of 178.86-fold (±14.02-fold, n = 9) CARiC9-20/19 CAR-T cell expansion (**Fig 2C**).

Among the healthy donors, we consistently observed a higher proportion of CD8^+^ cells (73.58% ± 12.71%, n = 9) compared to CD4^+^ cells (21.98% ± 12.31%, n = 9) among the CAR^+^ population **(Fig 2D)**. To further assess the phenotypic compositions of CARiC9-20/19 CAR-T cells, we determined the percentages of CD45RA^+^CD62L^+^CD95^-^ naïve T cell (T_N_), CD45RA^+^CD62L^+^CD95^+^ stem cell memory T cell (T_SCM_), CD45RA^-^CD62L^+^CD95^+^ central memory T cell (T_CM_), CD45RA^-^CD62L^-^CD95^+^ effector memory T cell (T_EM_), and CD45RA^-^CD62L^-^CD95^-^ effector T cell (T_EFF_) subsets in the CD4^+^ and CD8^+^ CAR^+^ populations. Compared to other T cell subsets, high percentages of CAR-T_SCM_ cells were observed in both the CD4^+^ (68.94% ± 9.13%, n = 9) and CD8^+^ (79.84% ± 8.19%, n = 9) populations **(Fig 2E)**. Furthermore, these CAR-T cells displayed low expression of exhaustion markers (PD-1, TIM-3, and LAG-3) and a senescence marker (CD57) at 10 days post-nucleofection **(Fig 2F and 2G)**. Interestingly, a small but significant population (9.92% ± 6.11%, n = 9) expressed the senescence marker KLRG-1.

**Fig 3:**
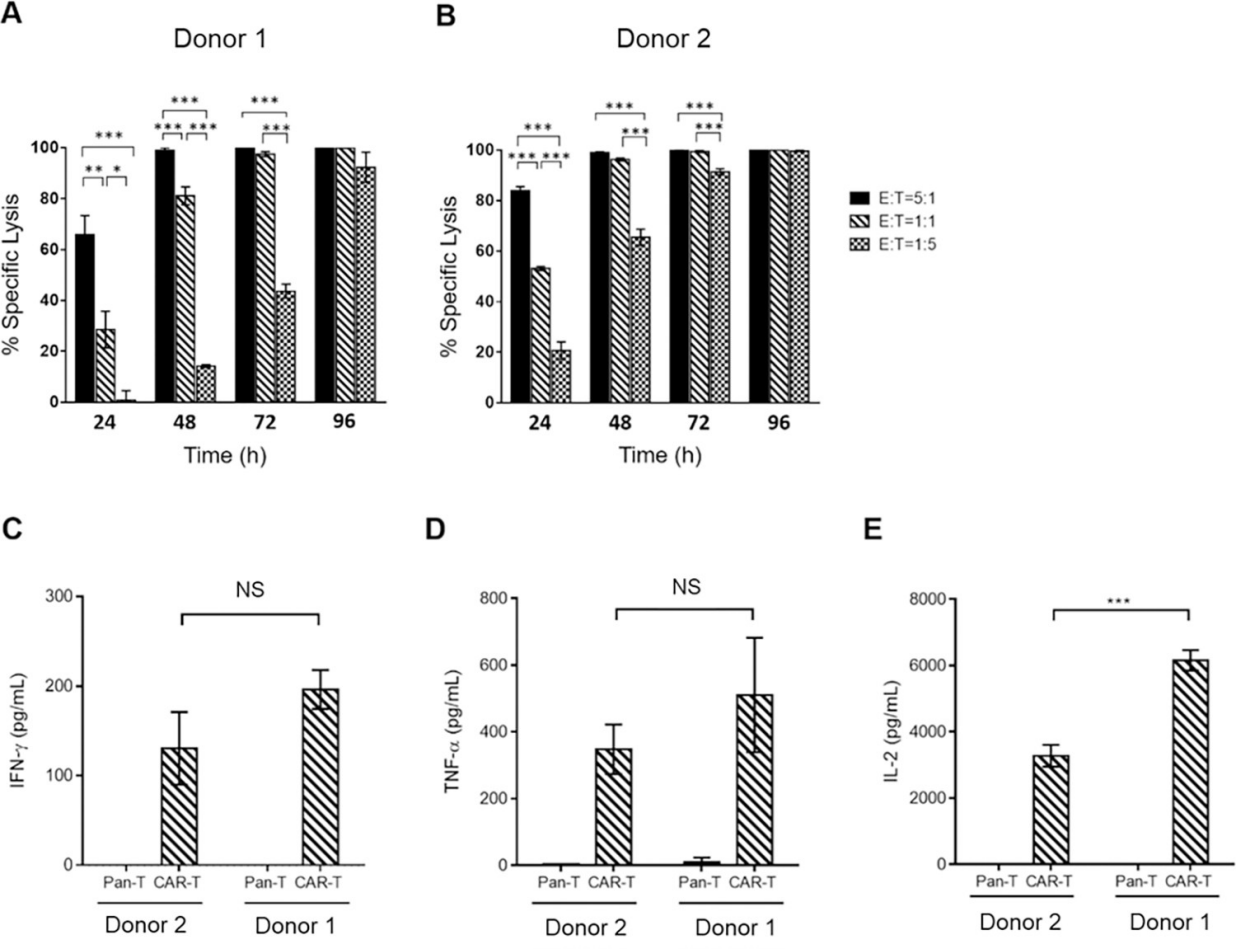
*In vitro* functional analysis of CARiC9-20/19 CAR-T cells. (A-B) CAR-T cells derived from two healthy donors were assessed for cytotoxicity against Raji-GFP/Luc cells by Celigo image cytometry. Groups were compared by One-way ANOVA with Tukey multiple comparison, ***p < 0.001, **p < 0.01, *p < 0.05. (C) IFN-γ, (D) TNF-α, and (E) IL-2 secretion by CARiC9-20/19 CAR-T cells following antigen stimulation was determined by ELISA. Pan-T cells (non-modified cells) served as a control group. Data represent mean ± SD, n = 3. Groups were compared by Student’s t-test, ***p < 0.001. NS, not statistically significant.

We next assessed the efficacy of CARiC9-20/19 CAR-T cells in Raji-GFP/Luc tumor and CARiC9-20/19 CAR-T cell co-cultures. While CARiC9-20/19 CAR-T cells lysed Raji-GFP/Luc tumors in a dose-dependent manner at early time points (24–48 h), complete tumor lysis was seen at 96 h **(Fig 3A and 3B)**. The CARiC9-20/19 CAR-T cells also lysed NALM-6/Luc tumors with similar kinetics **(S3 Fig)**. Furthermore, CARiC9-20/19 CAR-T cells secreted significant levels of pro-inflammatory cytokines IFN-γ, TNF-α and IL-2, as compared to pan-T cells **(Fig 3C and 3E)**. Collectively, these data suggest that functional CARiC9-20/19 CAR-T cells can be generated using *qPB*.

### CARiC9-20/19 CAR-T cells display enhanced and persistent anti-tumor function *in vivo*

**Fig 4:**
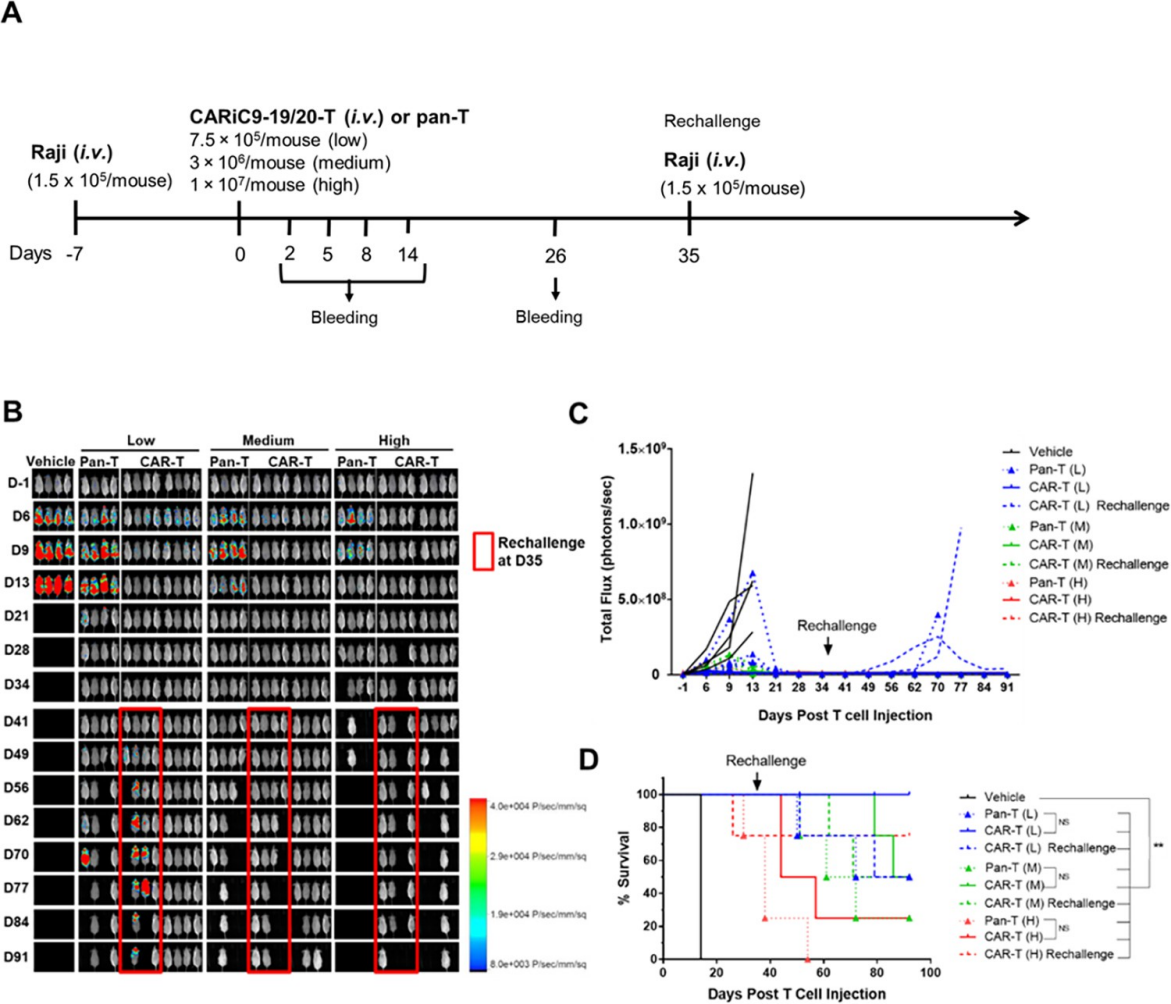
Anti-tumor activity of CARiC9-20/19 CAR-T cells in a B-cell lymphoma immunodeficient xenograft mouse model. (A) Schematic diagram of *in vivo* Raji xenograft model experimental design. (B-C) Bioluminescence imaging was performed to monitor tumor cell persistence (n = 4/group) and tumor growth were quantified by total flux (photon/s). (D) Kaplan-Meier survival curves for the different treatment groups are shown. **p < 0.01, *p < 0.05. NS, not statistically significant.

We next evaluated the anti-tumor efficacy of G-Rex cultured CARiC9-20/19 CAR-T cells in an *in vivo* xenotransplant immunodeficient mouse model. We injected 1.5 × 10^5^ Raji-GFP/Luc tumor cells *i*.*v*. followed by *i*.*v*. injection of 7.5 × 10^5^ (low dose), 3.0 × 10^6^ (medium dose), or 1 × 10^7^ (high dose) CARiC9-20/19 CAR-T cells 7 days later **(Fig 4A)**. As assessed by bioluminescence imaging, all mice in the vehicle group succumbed to tumors by day 21 **(Fig 4B and 4C)**. In contrast, Raji-GFP/Luc tumors were completely eradicated by day 13 in all three treatment groups. Anti-Raji activity was also observed in mice injected with non-transfected pan-T cells that were cultured under the same conditions. This graft-versus-tumor effect was observed beginning approximately two weeks following injection, which suggests it likely resulted from *in vivo* expansion of cells that recognize and kill the tumor cells via a CAR-independent, non-specific TCR-mediated mechanism. Graft-versus-tumor effect has also been observed by others in similar CAR-T xenograft tumor models [30–32]. While non-specific TCR-mediated killing may occur as a result of host-donor MHC mismatch, we would not expect such an anti-tumor effect in clinical autologous T-cell therapy. Cytokine release syndrome (CRS), associated with high levels of serum cytokines such as IL-6, is an important adverse effect in CAR-T therapy [5, 33]. In our study, the production of IL-6, IFN-γ, IL-2, and TNF-α remained at low or basal levels on days 2, 8 and 14 **(S4A Fig)**. In a clinical study on CD19-41BBz CAR, a similar lack of serum cytokine induction has been reported, suggesting low likelihood of severe CRS [34]. On day 26, no detectable levels of Raji-GFP/Luc tumors were observed in all three treatment groups **(S4B Fig)**. Moreover, residual CAR gene was observed in the blood of the medium and high dose groups **(S4C Fig)**.

While residual CARiC9-20/19 CAR-T cells could be detected up to day 26, it remained unclear whether these residual cells retained anti-tumor function. To address this question, we rechallenged half of the mice in each treatment group with 1.5 × 10^5^ Raji-GFP/Luc cells on day 35. Due to the lack of CAR-T cell persistence, most of the rechallenged mice in the low dose group eventually succumbed to tumors **(Fig 4B and 4C)**. In contrast, the rechallenged mice in the medium and high dose groups remained tumor-free throughout the experiment. Some mice in the medium and high dose groups were euthanized after meeting the criteria for euthanasia. The major symptoms of graft-versus-host disease (GvHD) after bone marrow transplantation are weight loss, ruffling of the fur, and hunched posture [35]. The euthanized mice from the medium and high dose groups likely suffered from GvHD, as severe fur ruffling and weight loss were observed. Of note, GvHD in the context of xenograft mouse models is a known major disadvantage of this experimental strategy [36, 37]. Most importantly, however, CARiC9-20/19 CAR-T treatment significantly improved the overall survival of tumor-bearing mice compared to vehicle control **(Fig 4D)**.

**Fig 5:**
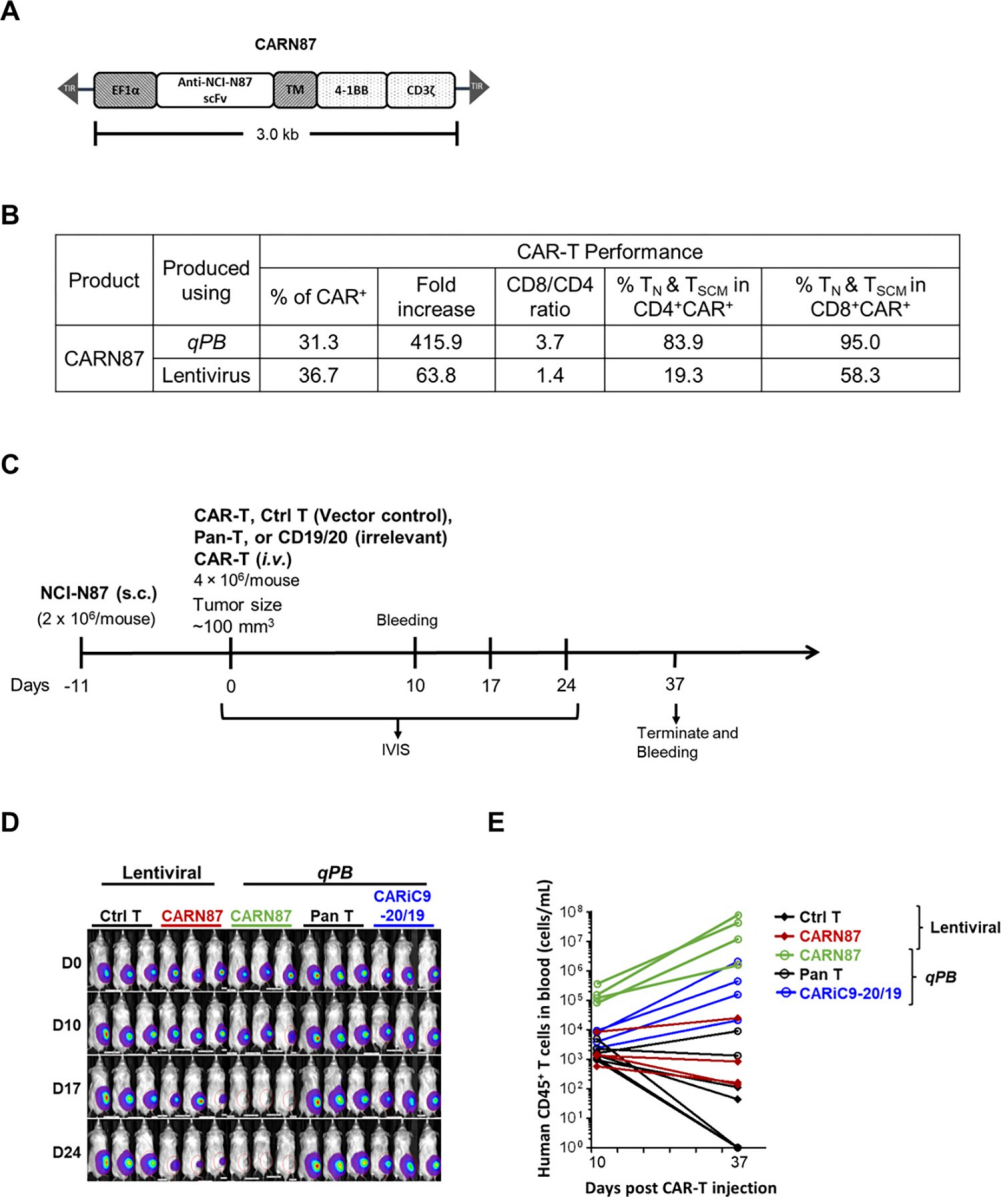
Anti-tumor activity of CARN87 cells in a gastric cancer immunodeficient xenograft mouse model. (A) Schematic diagram of the CARN87 CAR construct. (B) Performance of CAR-T cells produced using lentiviral and non-viral *qPB* cell production. (C) Schematic diagram of in vivo NCI-N87 xenograft model experimental design. (D) Bioluminescent imaging was performed to monitor tumor cell persistence (n = 3/group). (E) Human T cell counts of blood samples taken from the indicated group of mice at day 10 and day 37 post-CAR-T injection.

We next directly compared *qPB*-engineered CAR-T cells with CAR-T cells manufactured using a conventional viral vector. Given the large transgene size used to generate CARiC9-20/19 CAR-T cells (>5 kb), it would be difficult to express the same transgene in a viral vector system. Since this difficulty in transgene introduction would introduce additional variables that can potentially influence the results, we designed a construct that only carries a single CAR gene targeting a pan-cancer glycan antigen, designated as CARN87 **(Fig 5A)**. In an *in vivo* xenotransplant immunodeficient mouse model, we injected 2 × 10^6^ NCI-N87 tumor cells subcutaneously (*s*.*c*.) followed by 4 × 10^6^ lentivirus- or *qPB*-produced CARN87 CAR-T cells (*i*.*v*.) 11 days later. Mice injected with control T cells or pan-T cells served as negative controls. In addition, mice injected with *qPB*-produced CARiC9-20/19 CAR-T cells (do not target the NCI-N87 tumor cells) served as an additional control. We demonstrated that *qBT*-cultured *qPB*-produced CARN87 CAR-T cells were superior in performance compared to lentivirus-engineered counterparts, as indicated by their greater *in vitro* expansion capacity, higher CD8/CD4 ratio, and enrichment of CAR^+^ T_SCM_ and CAR^+^ T_N_ cells **(Fig 5B)**. We further compared the anti-tumor efficacy of CAR-T cells carrying CARN87 delivered using either *qPB* or a lentivirus approach in a NCI-N87 gastric carcinoma xenotransplant model **(Fig 5C)**. Since solid tumors are more challenging to eradicate, the differences in quality of the CAR-T cells produced using *qPB* and the lentivirus system in the NCI-N87 tumor model may be easier to differentiate. Complete tumor clearance was observed in *qPB*-produced CARN87 CAR-T cell-treated mice on day 17 but not in those treated with lentivirus-engineered CAR-T cells **(Fig 5D)**. Furthermore, unlike the lentivirus-engineered counterparts, circulating *qPB-*produced CAR-T cells proliferated robustly (10- to 100-fold) between days 10 and 37 **(Fig 5E)**. This difference may reflect the higher percentage of T_N_ and T_SCM_ cells present in *qPB*-produced CAR-T CD4 and CD8 populations **(Fig 5B)**.

### Functional CARiC9-20/19 CAR-T_SCM_ cells can be generated in sufficient numbers from cancer patients

**Fig 6:**
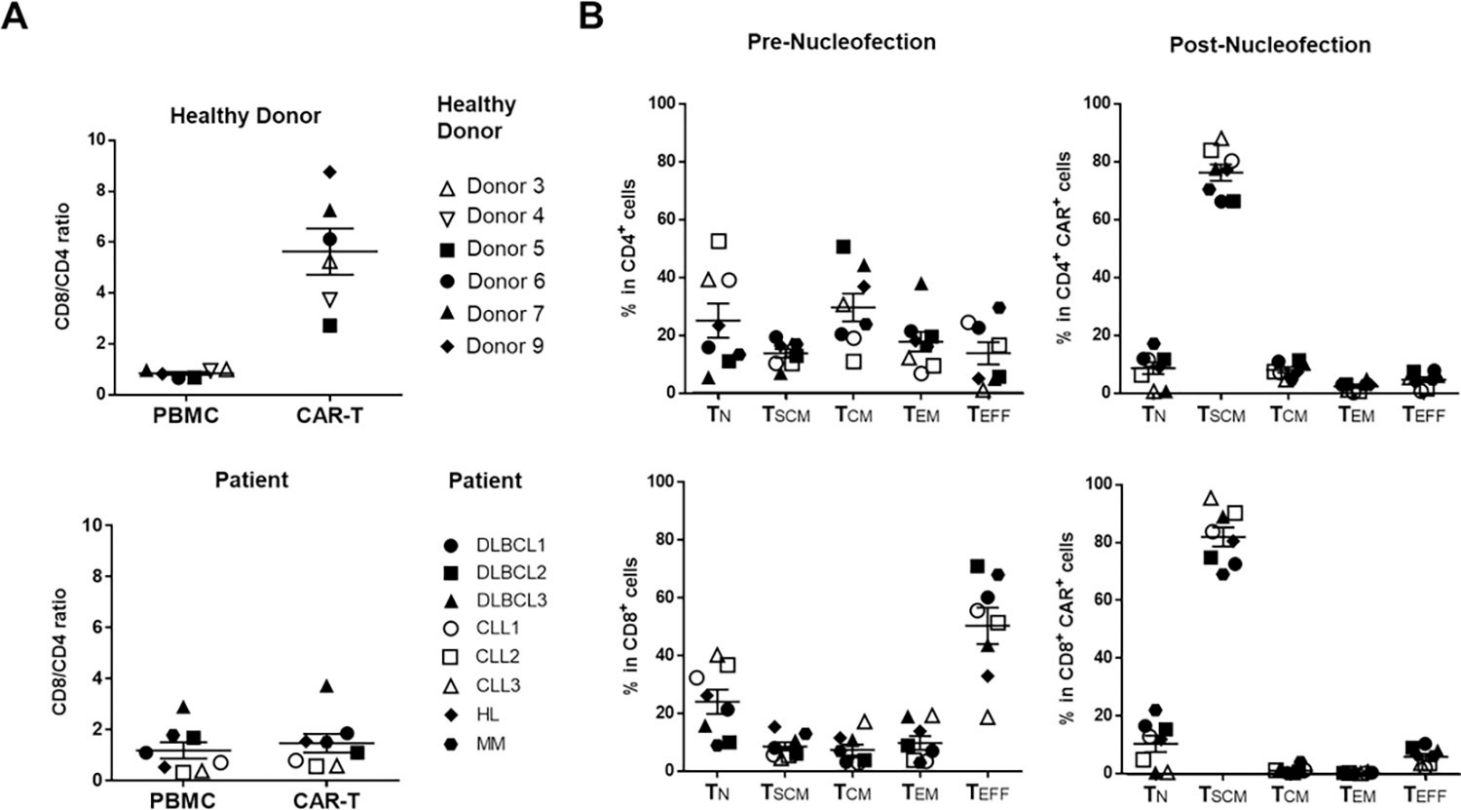
Assessment of cancer-patient-derived CARiC9-20/19 CAR-T cells. T cells derived from healthy donors and cancer patients were nucleofected with CARiC9-20/19. (A) CD8/CD4 ratios were assessed before (PBMC) and after (CAR-T) nucleofection. (B) Distribution of T_N_, T_SCM_, T_CM_, T_EM_, and T_EFF_ subsets in CD4^+^ (upper panel) and CD8^+^ (lower panel) CAR-T cell populations among the patient samples in (A). DLBCL, diffuse large B-cell lymphoma; CLL, chronic lymphocytic leukemia; HL, Hodgkin’s lymphoma; MM, multiple myeloma.

We next assessed whether sufficient numbers of high-quality CARiC9-20/19 CAR-T cells can be generated for clinical applications. In our experiments to this point, we had expanded all CARiC9-20/19 CAR-T cells with CD19-expressing aAPC, unless otherwise indicated. To streamline the manufacturing process, we tested whether we could still generate highly potent patient-derived CARiC9-20/19 CAR-T_SCM_ cells in the absence of aAPC selective expansion. We isolated PBMCs from six healthy donors, three diffuse large B-cell lymphoma (DLBCL) patients, three chronic lymphocytic leukemia (CLL) patients, one Hodgkin lymphoma (HL) patient, and one multiple myeloma (MM) patient to generate CARiC9-20/19 CAR-T cells. **Fig 6** and **Table 1** show summaries of the characteristics of the donor/patient-derived CAR-T cells.

**Table 1:** Comparison of CARiC9-20/19 CAR-T cells generated from healthy donors and cancer patients.

Disease		Healthy Donor	Patients	DLBCL1	DLBCL2	DLBCL3	CLL1	CLL2	CLL3	HL	MM
**Copy Number**		1.85–3.47	1.82–5.46	2.51	5.46	2.21	3.36	2.81	3.76	2.04	1.82
**% of CAR** ^**+**^		32.5–49.6	19.93–53.22	26.33	50.01	34.23	40.64	33.41	53.22	26.24	19.93
**Fold Expansion**		43.54–157.55	57.95–198.96	67.22	67.30	60.38	104.57	57.95	198.96	149.75	139.35
**Cell Number (x10** ^**6**^ **)**		17.34–50.60	4.58–27.54	7.29	4.95	5.80	15.76	7.71	4.58	24.08	27.54
**% of B Cells (CD19** ^**+**^ **)**		0.01–0.36	0–1.18	0.77	1.18	0.35	0	0	0.61	0	0
**% of NK Cells (CD3** ^**-**^ **, CD56** ^**+**^ **)**		0.04–0.27	0.02–2.69	0.06	2.69	0.63	0.03	0.02	0.02	0.08	0.05
**% of Exhaustion Marker**^**+**^ **Cells**	**PD-1**	0.40–1.96	0.96–7.80	5.35	4.15	4.16	0.96	2.42	6.01	1.70	7.80
	**TIM-3**	0.10–5.34	1.07–2.96	1.77	2.96	1.98	1.40	2.32	2.37	1.35	1.07
	**LAG-3**	0.90–7.00	2.36–22.82	9.88	12.38	22.82	3.24	4.03	2.55	2.36	2.56
**% of Senescence Marker**^**+**^ **Cells**	**KLRG-1**	2.24–14.61	2.83–14.70	11.80	14.70	8.20	2.83	4.15	6.06	4.05	5.33
	**CD57**	0.03–0.68	0.63–5.82	5.82	1.46	2.54	1.11	1.78	0.63	1.13	1.28
**Potency (% of Raji cell lysis)** **(fresh cells, E/T = 5, 48 h)**		89.74–95.38	98.96–99.88	99.66	99.7	99.7	99.88	99.45	99.07	98.96	99.42
**Potency (% of Raji cell lysis)** **(frozen cells, E/T = 5, 48 h)**		44.99–61.96	45.46–84.31	73.35	74.21	75.21	84.31	75.70	45.46	77.49	70.31

CAR-T cells generated from PBMCs of 6 healthy donors and 8 cancer patients were cultured in 24-well G-Rex for 10 days. The cell number of 24-well G-Rex culture is 50-times 50 less than a 1 L G-Rex culture (2.29×10^8^–1.38×10^9^ cells in 1 L G-Rex). DLBCL, diffuse large B-cell lymphoma; CLL, chronic lymphocytic leukemia; HL, Hodgkin lymphoma; MM, multiple myeloma.

In 10 days, we expanded the CAR-T cells to 4.58 × 10^6^–2.76 × 10^7^ cells in a 24-well G-Rex culture, which is equivalent to a clinically relevant number of 2.29 × 10^8^–1.38 × 10^9^ cells in a 1 L G-Rex culture vessel. The measured values of CAR expression (19.9–53.22%, n = 8) and fold expansion (58.0–198.96-fold, n = 8) for patient cells were comparable to those from healthy donors (32.5–49.6% and 43.54–157.55-fold, n = 6) **(Table 1)**. CAR-T purity was confirmed by the low percentage of CD19^+^ B cells and CD3^-^CD56^+^ NK cells in both healthy donors (0.01–0.36% B cells; 0.04–0.27% NK cells, n = 6) and patients (0–1.18% B cells; 0.02–2.69% NK cells, n = 8) **(Table 1)**. The proportions of CD8^+^ and CD4^+^ cells were balanced in both the healthy donors (CD8/CD4 = 0.85 ± 0.15, n = 6) and patients (CD8/CD4 = 1.17 ± 0.9, n = 8) prior to nucleofection **(Fig 6A)**. After nucleofection, donor-derived CAR-T cells had higher CD8/CD4 ratios (5.64 ± 2.23, n = 6). However, only some patients had increase CAR^+^ CD8 T cell population **(Fig 6A)**. Notably, compared to pre-nucleofection (PBMC) levels, the percentages of T_SCM_ subsets at 10 days post-nucleofection increased in all patients among CD4 (from 13.75% ± 4.25% to 76.25% ± 7.99%, n = 6) and CD8 (from 8.62% ± 3.96% to 81.90% ± 9.35%, n = 6) populations, confirming the ability of our *qPB*-based CAR-T production system to support T_SCM_ differentiation **(Fig 6B,** see **S5 Fig** for gating strategy). Low percentages of CARiC9-20/19 CAR-T cells expressed the exhaustion markers PD-1 and TIM-3 and senescence marker CD57 at 10 days post-nucleofection **(Table 1)**. In addition, the percentage of cells expressing the exhaustion marker LAG-3 at 10 days post-nucleofection was low in CAR-T cultures derived from CLL, HL, and MM patients (2.4–4%, n = 5), but it was slightly elevated in cells derived from DLBCL patients (9.9–22.8%, n = 3). While KLRG-1 expression (7.28% ± 4.83%, n = 6) was detectable in CARiC9-20/19 CAR-T patient cells, it was significantly lower than KLRG-1 expression before nucleofection (21.27% ± 9.37%, n = 6; *p* = 0.0087) **(S6A Fig)**. The decrease in KLRG-1 expression was especially prominent in the CD8^+^ population (52.50% ± 14.51% vs. 14.59% ± 10.15%, n = 6; *p* = 0.0004) **(S6B Fig)**. In a 48-h cytotoxicity assay, we further confirmed that CARiC9-20/19 CAR-T cells derived from both healthy donors and patients could eradicate Raji tumors **(Table 1)**. Overall, these results suggest that *qPB*-based CAR-T engineering system is a promising technology for generating functional CAR-T_SCM_ cells for cancer immunotherapy applications.

## Discussion

One strategy to overcome challenges faced by solid cancers is to engineer T cells to simultaneously express multiple therapeutic genes for enhanced functionality. *qPB* can produce CAR-T cells with transgene size >7.6 kb in a single cargo, which is not achievable by conventional lentiviral/retroviral CAR-T engineering. The limited cargo capacity of lentiviral/retroviral vectors may account for the lack of current CAR-T clinical trials targeting B-cell malignancies with both dual-targeting CARs and incorporating an iCasp9 safety switch gene (total 5.2 kb transgene size). In this report, we present evidence supporting the feasibility of manufacturing potent iCasp9-regulatable CD20/CD19-targeted CAR-T_SCM_ cells using *qPB* and culture with *qBT* for clinical application. We have demonstrated the purity, potency and consistent profiles of CAR-T cells manufactured using the *qPB* system.

Random gene integration in CAR-T therapy is a major safety concern, as some patients in a recent clinical study developed CAR-T-cell lymphoma following *piggyBac*-modified CAR19 therapy [38]. A mechanistic study on this topic revealed a high number of *piggyBac* integrants, but none were inserted into or near typical oncogenes [39]. Nevertheless, this study highlights the importance of keeping a low integrant copy number per genome for gene therapy products. By adjusting the total DNA and the *qPB* donor to helper DNA ratio, we can further lower the copy number to <5 copies per CAR^+^ cell without compromising yield quality and quantity (data not shown).

Acute toxicities such as CRS and ICANS have also been major concerns in CAR-T therapy-treated patients [11, 12, 40]. To safely manage toxicities, CAR-T cells can be eradicated by triggering death of CAR-T cells (e.g., anti-EGFRt antibody) via antibody-dependent cellular cytotoxicity (ADCC) or by using a small molecule that activates a suicide gene introduced in the CAR design (e.g., iCasp9) [40]. Triggering ADCC has a slower onset to killing than activation of a suicide gene. We included an iCasp9 safety switch in our CARiC9-20/19 CAR-T design so that we would be able to mitigate adverse effects of CRS and ICANS within hours. Indeed, Foster *et al*. has recently demonstrated that acute ICANS can be effectively controlled by treating CAR-T therapy patients with the iCasp9-activating agent, rimiducid (AP1903) [41].

In this study, we also addressed challenges associated with manufacture of CAR-T cells by testing the relative contributions of *qBT* and aAPC. While we used aAPC in our initial experiments, we could generate sufficient quantities of patient-derived CARiC9-20/19 CAR-T cells without aAPC-induced enrichment by culturing cells with *qBT* alone. The use of *qBT* highly enriched CD4^+^ T_SCM_ populations, which may be critical for maintaining CD8^+^ CAR-T cells at the T_SCM_ differentiation stage. We also confirmed that *qPB*-engineered CAR-T cells could effectively control tumors without aAPC enrichment. Furthermore, we demonstrated that 2.29 × 10^8^–1.38 × 10^9^ T cells could be generated in a 1-L G-Rex culture within a short time-frame of 10 days using cancer patient-derived T cells. Even at a low CAR^+^ percentage of 20%, sufficient numbers of CAR^+^ CAR-T cells (4.58 × 10^6^–2.76 × 10^7^) can still be produced for treatment, and CAR^+^ T cells could easily be enriched with aAPC should the need arise.

The high potency of CARiC9-20/19 CAR-T cells can be attributed to the balanced CD8/CD4 ratio among CAR-T cells and the high enrichment of T_SCM_ in the engineered cells. It has been reported that a balanced CD8/CD4 CAR-T ratio results in high remission rates [42, 43]. To achieve this balance, others have engineered CD4^+^ and CD8^+^ CAR-T cells separately and combined the cultures for adoptive transfer [42]. While *qPB*-engineered CAR-T cells derived from healthy donors generally exhibited CD8 enrichment, cancer patient-derived CAR-T cells had a balanced CD8/CD4 CAR-T ratio (**Fig 6**). Thus, *qPB* naturally achieves the desired CD8/CD4 balance without the need to engineer CD4 and CD8 CAR-T cells separately, as in SB100X-modified CAR-T clinical trials [44]. In a solid tumor NCI-N87 xenograft model, we further demonstrated superior performance, anti-tumor efficacy, and *in vivo* expansion of *qPB*-produced CAR-T cells over lentivirus-produced CAR-T cells. Overall, these observations suggest that *qPB*-produced CAR-T cells are potent.

One approach to harness the potency of less-differentiated T cells such as T_SCM_ is to shorten the *ex vivo* expansion time of CAR-T product. Ghassemi *et al*. demonstrated that shortening the lentiviral CAR-T manufacturing process to 24-hours resulted in a higher percentage of CAR-T cells with a memory phenotype [45]. Although these CAR-T cells displayed stronger *in vivo* anti-tumor activity than conventional counterparts, successful clinical translation of this process remains to be demonstrated. Another approach is to culture CAR-T cells with specific cytokines or chemical reagents [18, 19, 46–48]. Additionally, CAR-T cell production from defined T cell subsets has been shown to be beneficial in enriching the T_SCM_ population [49]. However, none of these approaches can enrich T_SCM_ populations to a level similar to that achieved by our *qPB*-based system. While T_SCM_ cells make up a small percentage of T cells derived from cancer patients, genetically modifying these cells using *qPB* followed by CAR-T cell expansion with *qBT* resulted in enrichment of T_SCM_ populations as high as >80% in both the CD4^+^ and CD8^+^ CAR^+^ T cell populations. Furthermore, we observed low expression of exhaustion (PD-1, TIM-3 and LAG-3) and senescence (CD57) biomarkers in CARiC9-20/19 CAR-T cells at 10 days post-nucleofection. While a significant population of CARiC9-20/19 CAR-T cells expressed the exhaustion marker KLRG-1, this proportion was significantly lower than that seen in freshly isolated PBMCs. Using a conditional knockout mouse model to map the fate of T cells, Herndler-Brandstetter *et al*. described a class of memory T cell population developed from KLRG-1^+^ effector CD8 T cells that have lost KLRG-1 expression [15]. These “exKLRG-1 memory cells” have been demonstrated to inhibit tumor growth more efficiently than KLRG-1^+^ cells in an OT-I melanoma mouse model. T_SCM_ cells generally lack KLRG-1 expression, and KLRG-1^+^ T_SCM_ cells have been associated with cancer patients undergoing relapse [16, 17]. Given the significant decrease in KLRG-1 expression in CARiC9-20/19 CAR-T cells, it is possible that a significant proportion of CAR-T_SCM_ cells in our model were actually exKLRG-1 memory cells. Thus, one potential mechanism that may contribute to the ability of *qPB* to generate highly potent CAR-T_SCM_ cells may be the conversion of KLRG-1^+^ effector CD8^+^ T cells to exKLRG-1 memory cells.

One major challenge of CAR-T manufacturing is the variation and inconsistency of CAR-T cell products [18]. In one study of ten CLL and ALL patients, CAR-T cells expanded 23.6-385-fold with retroviral CAR transduction of 4–70% [19]. Lentiviral transduction rates of 5.5–45.3% were reported by other studies of CLL and ALL patients [45, 46]. In our CARiC9-20/19 CAR-T study, we observed a tighter range of CAR^+^ percentages (19.9–53.22%) and expansion (58.0- to 198.96-fold) among the cells derived from eight cancer patients **(Table 1**). Most importantly, our data showed that CARiC9-20/19 CAR-T cells derived from both healthy donors and patients could effectively eradicate tumor cells.

Allogeneic CAR-T therapy has been expected to be an inevitable replacement of autologous CAR-T products since the first allogenic CAR-T clinical study in 2016 [50]. However, clinical data suggest allogeneic CAR-T products are less potent and have limited CAR-T cell persistence. Induced pluripotent stem cells (iPSC)-derived CAR-T cells may be a promising alternative for allogeneic CAR-T therapy, but the heavy mutational burden in iPSCs also poses safety concerns for clinical applications. Thus, autologous CAR-T therapy will likely remain the mainstay of CAR-T-based treatment in the short to medium term. In this regard, we have demonstrated that our *qPB*-based CAR-T production system can address many of the current challenges of autologous CAR-T therapy.

In summary, *qPB* is an efficient system for the manufacturing of safe, potent and cost-effective CAR-T products. We demonstrated that *qPB* could be used to produce patient-derived CARiC9-20/19 CAR-T cells with many desirable attributes, including a high proportion of CAR^+^ T_SCM,_ balanced CD8/CD4 ratio, low exhaustion and senescence marker expression, high expansion capacity, and high anti-tumor efficacy. The simplicity and robustness of our strategy to create multiplex CAR-T cells using *qPB* may help to improve accessibility of CAR-T therapy and also has potential to allow for the generation of armored CAR-T therapies to treat solid tumors in the future.

## Supporting information

S1 Fig Comparison of perfusion and fed-batch-cultured CAR-T cells. (A) Characterization of CARiC9-20/19 CAR-T cells cultured in conventional plates (perfusion) or G-Rex (fed batch) at day 10 after nucleofection. (B) Cytolytic activities of CAR-T cells after 48 or 72 h of co-culture with Raji-GFP/Luc target cells (E:T ratio of 5:1) were assessed by Celigo image cytometry. (A-B) Data represent mean ± SD for 6 healthy donors, n = 6. *p < 0.05, **p < 0.01, ***p < 0.001.
(TIF)

S2 Fig Safety assessment of CARiC9-20/19 CAR-T cells. (A) Elimination of CARiC9-20/19 CAR-T cells after iCasp9 activation. Percentage of CAR^+^ T cells was assessed by flow cytometry 24 h after AP1903 treatment. Data represent mean ± SD mean, n = 3 (One-way ANOVA with Tukey multiple comparison). *p < 0.05, **p < 0.01, ***p < 0.001. (B) Average CAR copy numbers were assessed in CARiC9-20/19 CAR-T cell products following electroporation with different amounts of DNA using primers against the scFv CAR region by ddPCR, n = 2 or 3.
(TIF)

S3 Fig *In vitro* functional analysis of CARiC9-20/19 CAR-T cells. CAR-T cells derived from one healthy donor was assessed for cytotoxicity against Raji-GFP/Luc cells or NALM-6-GFP/Luc by Celigo image cytometry. Groups were compared by One-way ANOVA with Tukey multiple comparison, ***p < 0.001, **p < 0.01, *p < 0.05.
(TIF)

S4 Fig Detection of CARiC9-20/19 CAR-T cell cytokine secretion in a B-cell lymphoma immunodeficient xenograft mouse model. (A) Plasma from mice were collected on days 2, 5, 8, and 14 after CAR-T cell administration and analyzed for IFN-γ, TNF-α, IL-2, and IL-6 by ELISA. Copy numbers of (B) luciferase and (C) CAR were determined in mouse blood samples on day 26 after CAR-T cell administration to monitor the presence of CAR^+^ T cells and Raji-GFP/Luc cells, respectively, in the mouse circulation (n = 8 for CAR-T (L) and n = 7 for CAR-T (M) and CAR-T (H)). Dotted lines represent the detection limits of qPCR.
(TIF)

S5 Fig Gating strategy for T_SCM_ cells. CD4 or CD8 T_SCM_ phenotyping and quantification strategy for analysis. (A) PBMCs were performed by sequential gating on (1) lymphocytes without debris, (2) PI^-^ or 7AAD^-^ (live) cells, (3) CD3^+^ cells, (4) either CD4^+^ or CD8^+^ cells, (5) CD45RA^+^ and CD62L^+^ cells, and (6) CD95^+^ cells. (B) CAR-T cells were analyzed as in (A), except in step (2) PI^-^ or 7AAD^-^ (live) and CAR^+^ cells are gated. Shown are representative data plots of patient DLBCL3.
(TIF)

S6 Fig Comparison of exhaustion and senescence before and after CAR nucleofection. (A) Expression of exhaustion (PD-1, TIM-3, and LAG-3) and senescence (KLRG-1 and CD57) markers in cells from cancer patients in Table 1 before (PBMC) and after (CAR-T) nucleofection. (B) Expression of KLRG-1 within the CD8 population of the cancer patients. * *p* < 0.05, ** *p* < 0.01, *** *p* < 0.001. NS, not statistically significant.
(TIF)

S1 Data set (DOCX)

## List of abbreviations

aAPC: artificial antigen presenting cells
CAR: chimeric antigen receptor
CLL: chronic lymphocytic leukemia
CRS: cytokine release syndrome
CT: threshold cycle
DLBCL: diffuse large B-cell lymphoma
EFS: event-free survival
CARiC9-20/19: bicistronic CD20/CD19-targeted iCasp9 regulatable CAR-T cells
GMP: good manufacturing practice
GVHD: graft-versus-host disease
HL: Hodgkin lymphoma
ICANS: immune effector cell-associated neurotoxicity syndrome
iCasp9: inducible caspase 9
i.v.: intravenously
MM: multiple myeloma
NHL: Non-Hodgkin lymphoma
PBMCs: peripheral blood mononuclear cells
PE: R-phycoerythrin
PFS: progression-free survival
qPB: *Quantum*
*pBac*; T_CM_: central memory T cell
T_EFF_: effector T cell
T_N_: naïve T cell
T_SCM_: T stem cell memory
