# Supplementary figures and images for: Manufacturing CD20/CD19-targeted iCasp9 regulatable CAR-TSCM cells using a Quantum pBac-based CAR-T engineering system
Source: PLoS One. 2024 Aug 27;19(8):e0309245. doi: 10.1371/journal.pone.0309245 (PMC11349195; doi:10.1371/journal.pone.0309245)

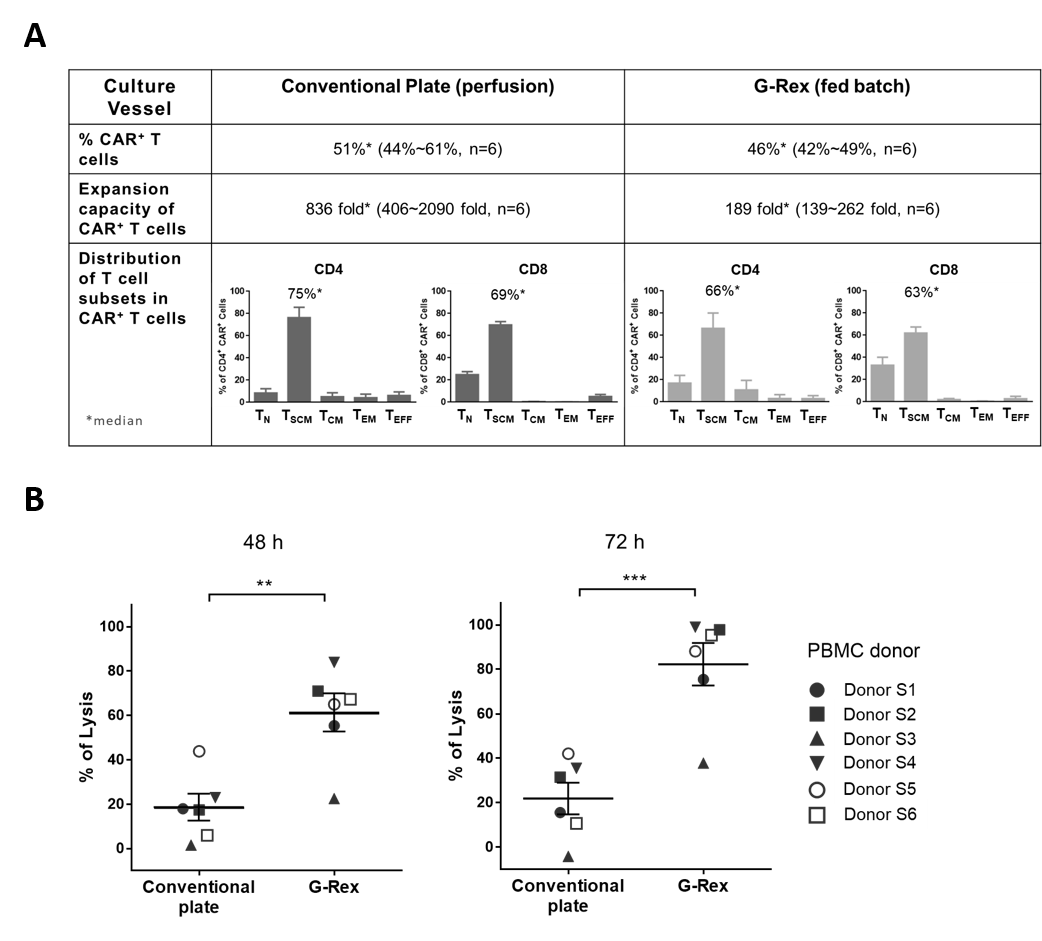

Supplement: S1 Fig — (A) Characterization of CARiC9-20/19 CAR-T cells cultured in conventional plates (perfusion) or G-Rex (fed batch) at day 10 after nucleofection. (B) Cytolytic activities of CAR-T cells after 48 or 72 h of co-culture with Raji-GFP/Luc target cells (E:T ratio of 5:1) were assessed by Celigo image cytometry. (A-B) Data represent mean ± SD for 6 healthy donors, n = 6. *p < 0.05, **p < 0.01, ***p < 0.001. (TIF) [file pone.0309245.s001.tif]

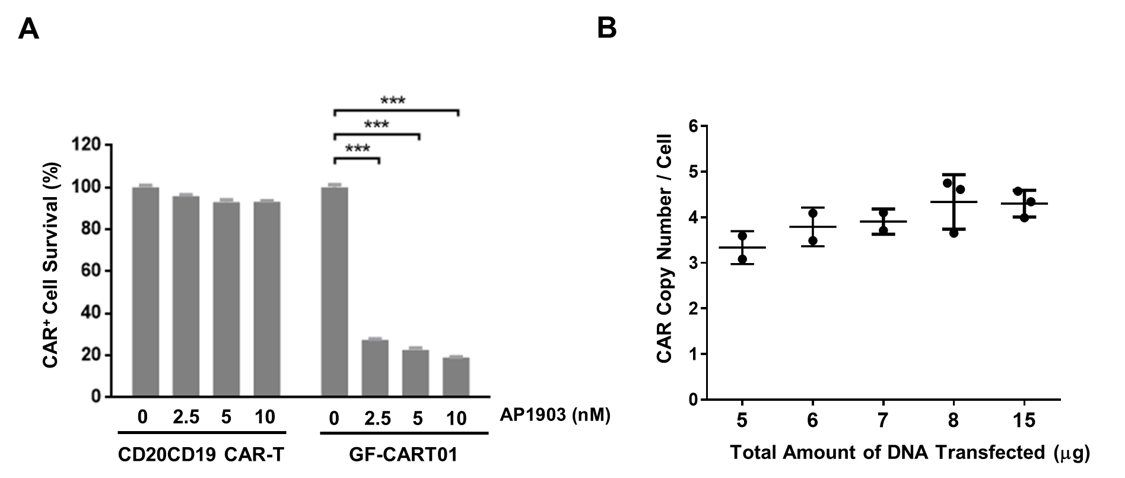

Supplement: S2 Fig — (A) Elimination of CARiC9-20/19 CAR-T cells after iCasp9 activation. Percentage of CAR+ T cells was assessed by flow cytometry 24 h after AP1903 treatment. Data represent mean ± SD mean, n = 3 (One-way ANOVA with Tukey multiple comparison). *p < 0.05, **p < 0.01, ***p < 0.001. (B) Average CAR copy numbers were assessed in CARiC9-20/19 CAR-T cell products following electroporation with different amounts of DNA using primers against the scFv CAR region by ddPCR, n = 2 or 3. (TIF) [file pone.0309245.s002.tif]

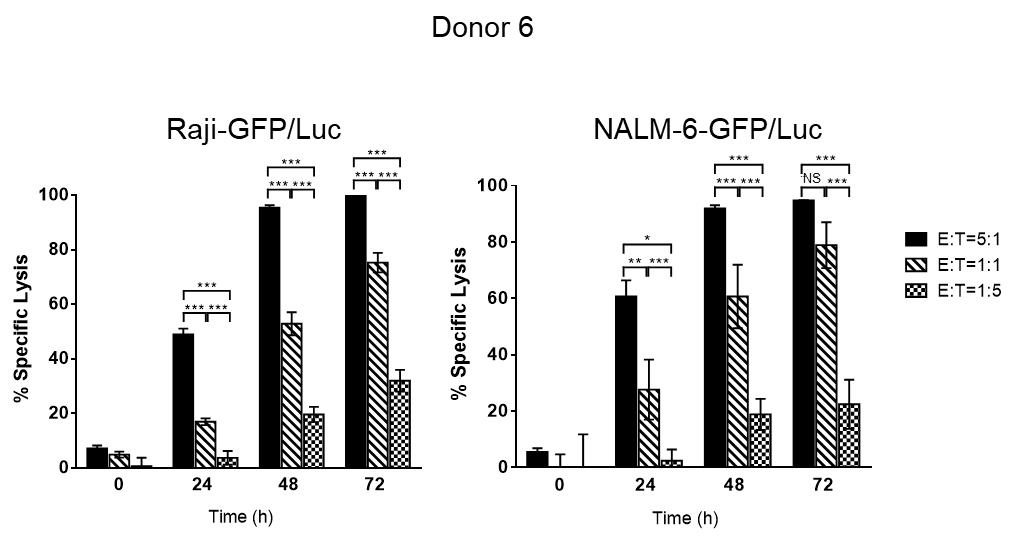

Supplement: S3 Fig — CAR-T cells derived from one healthy donor was assessed for cytotoxicity against Raji-GFP/Luc cells or NALM-6-GFP/Luc by Celigo image cytometry. Groups were compared by One-way ANOVA with Tukey multiple comparison, ***p < 0.001, **p < 0.01, *p < 0.05. (TIF) [file pone.0309245.s003.tif]

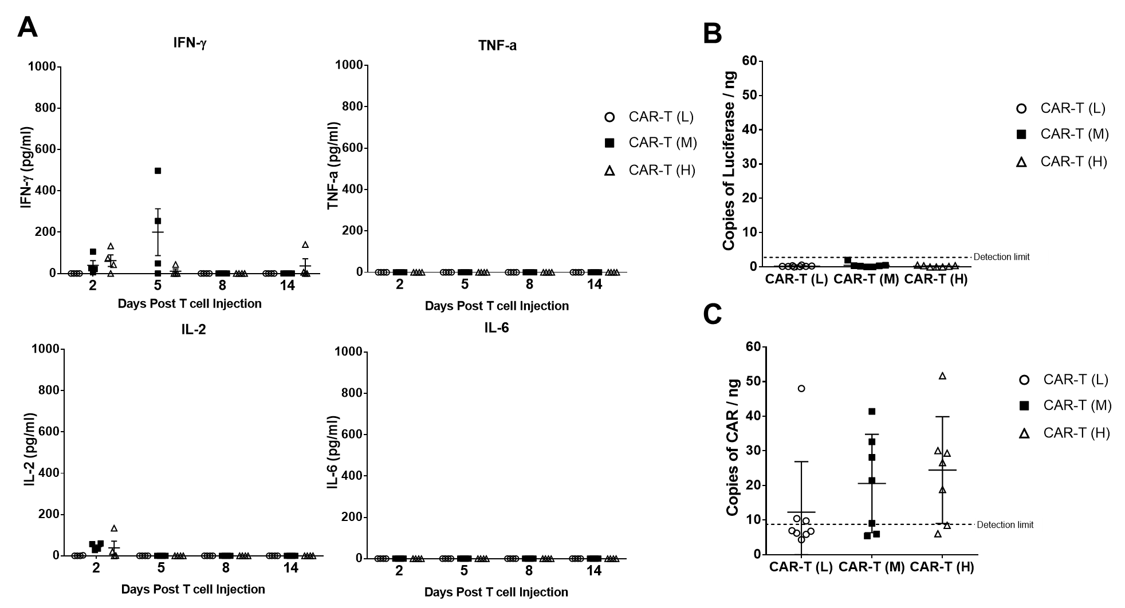

Supplement: S4 Fig — (A) Plasma from mice were collected on days 2, 5, 8, and 14 after CAR-T cell administration and analyzed for IFN-γ, TNF-α, IL-2, and IL-6 by ELISA. Copy numbers of (B) luciferase and (C) CAR were determined in mouse blood samples on day 26 after CAR-T cell administration to monitor the presence of CAR+ T cells and Raji-GFP/Luc cells, respectively, in the mouse circulation (n = 8 for CAR-T (L) and n = 7 for CAR-T (M) and CAR-T (H)). Dotted lines represent the detection limits of qPCR. (TIF) [file pone.0309245.s004.tif]

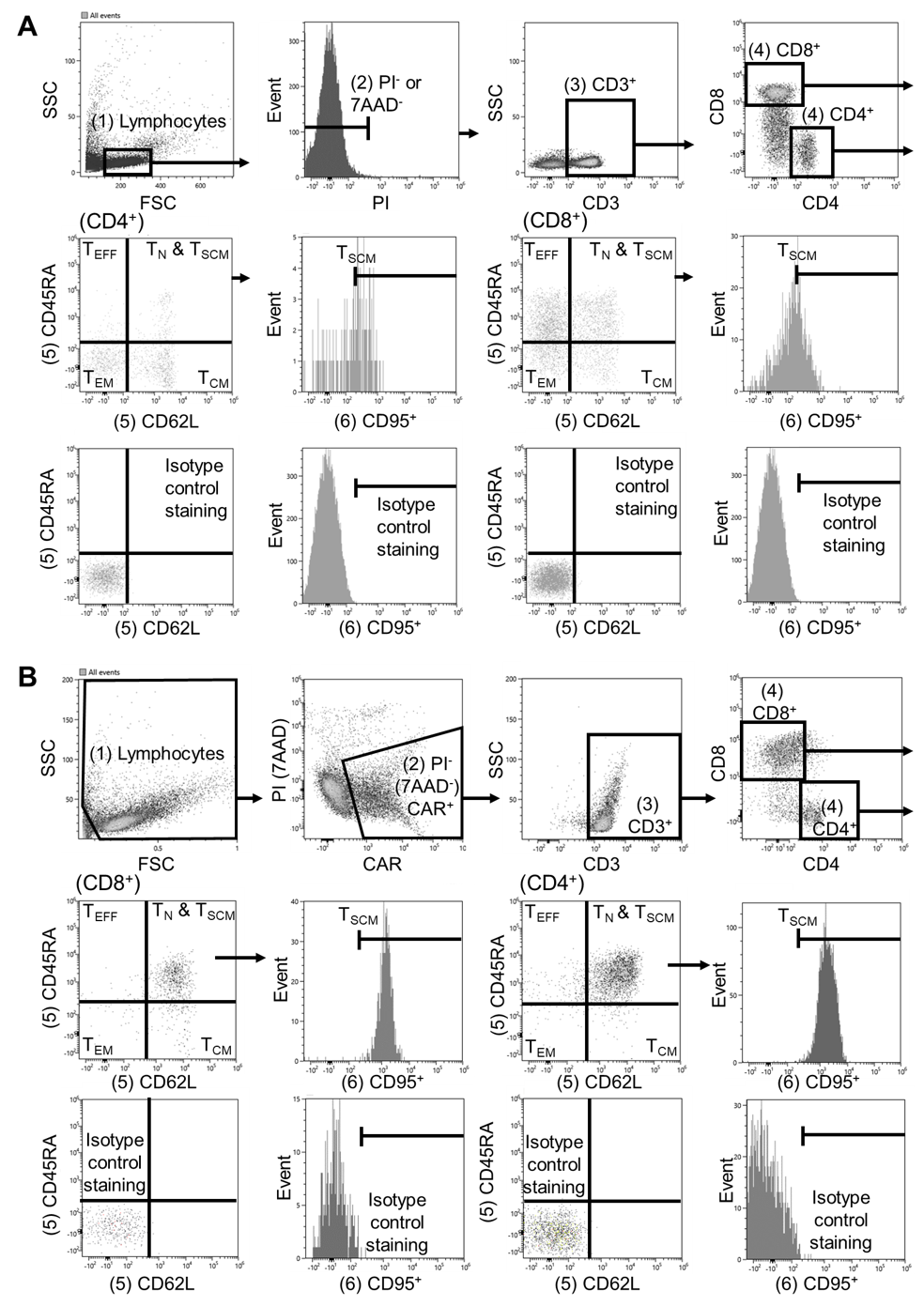

Supplement: S5 Fig — CD4 or CD8 TSCM phenotyping and quantification strategy for analysis. (A) PBMCs were performed by sequential gating on (1) lymphocytes without debris, (2) PI- or 7AAD- (live) cells, (3) CD3+ cells, (4) either CD4+ or CD8+ cells, (5) CD45RA+ and CD62L+ cells, and (6) CD95+ cells. (B) CAR-T cells were analyzed as in (A), except in step (2) PI- or 7AAD- (live) and CAR+ cells are gated. Shown are representative data plots of patient DLBCL3. (TIF) [file pone.0309245.s005.tif]

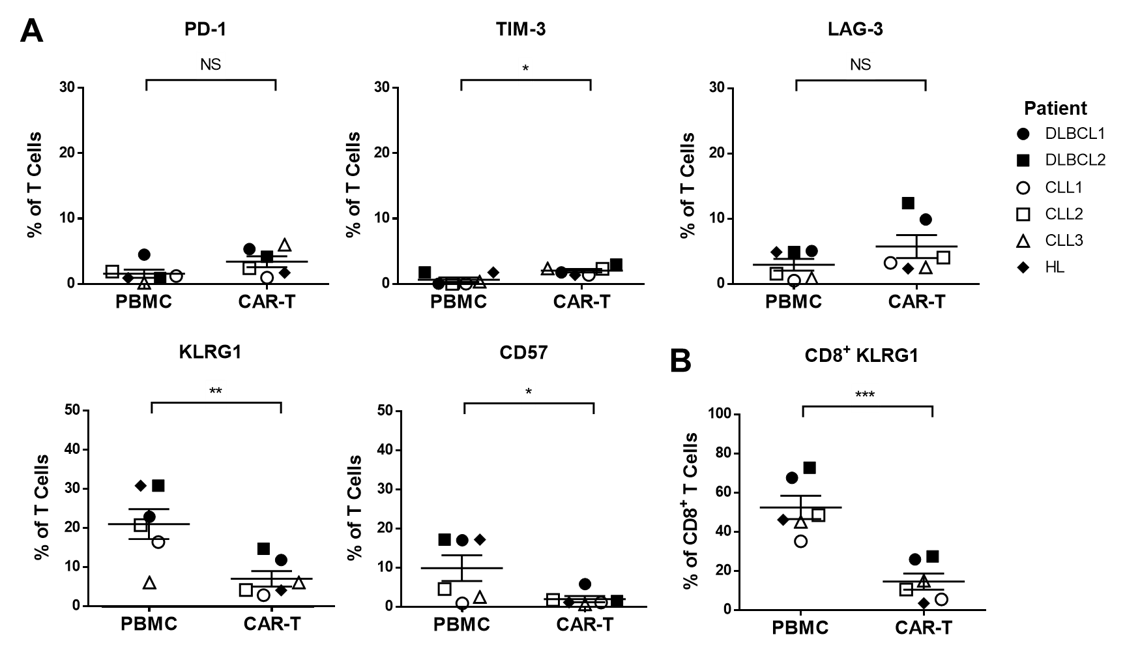

Supplement: S6 Fig — (A) Expression of exhaustion (PD-1, TIM-3, and LAG-3) and senescence (KLRG-1 and CD57) markers in cells from cancer patients in Table 1 before (PBMC) and after (CAR-T) nucleofection. (B) Expression of KLRG-1 within the CD8 population of the cancer patients. * p < 0.05, ** p < 0.01, *** p < 0.001. NS, not statistically significant. (TIF) [file pone.0309245.s006.tif]
