# Supplementary material for: Manufacturing CD20/CD19-targeted iCasp9 regulatable CAR-TSCM cells using a Quantum pBac-based CAR-T engineering system
Source: PLoS One. 2024 Aug 27;19(8):e0309245. doi: 10.1371/journal.pone.0309245 (PMC11349195; doi:10.1371/journal.pone.0309245)
Supplement: S1 Data set — (DOCX) [file pone.0309245.s007.docx]

**Supporting Information Dataset**

| **Figure 1B** | Fold change | Donor S9 | Donor S10 | Donor S11 | Donor S12 | Donor S13 | Donor S14 | Mean | SD |
| --- | --- | --- | --- | --- | --- | --- | --- | --- | --- |
|  | -qBT -aAPC | 7.29 | 3.25 | 4.07 | 7.05 | 57.74 | 2.18 | 13.60 | 21.72 |
|  | -qBT +aAPC | 21.69 | 13.89 | 13.93 | 28.79 | 154.51 | 4.67 | 39.58 | 56.89 |
|  | +qBT -aAPC | 74.84 | 110.83 | 53.91 | 60.81 | 176.51 | 51.44 | 88.06 | 48.51 |
|  | +qBT +aAPC | 195.28 | 518.36 | 291.42 | 172.82 | 557.81 | 183.13 | 319.80 | 174.74 |
|  |  |  |  |  |  |  |  |  |  |
| **Figure 1C** | %CAR^+^ live cells | Donor S9 | Donor S10 | Donor S11 | Donor S12 | Donor S13 | Donor S14 | Mean | SD |
|  | -qBT -aAPC | 17.16 | 21.02 | 28.43 | 38.03 | 34.34 | 28.12 | 27.85 | 7.84 |
|  | -qBT +aAPC | 30.52 | 27.48 | 21.38 | 40.48 | 46.44 | 21.55 | 31.31 | 10.22 |
|  | +qBT -aAPC | 51.39 | 61.40 | 60.28 | 51.70 | 41.82 | 44.73 | 51.89 | 7.92 |
|  | +qBT +aAPC | 70.42 | 71.65 | 76.80 | 73.41 | 66.65 | 64.96 | 70.65 | 4.36 |
|  |  |  |  |  |  |  |  |  |  |
| **Figure 1D** | % T_SCM_ of CD4 T cells | Donor S9 | Donor S10 | Donor S11 | Donor S12 | Donor S13 | Donor S14 | Mean | SD |
|  | -qBT -aAPC | 37.69 | 49.04 | 54.92 | 47.10 | 50.40 | 35.42 | 45.76 | 7.62 |
|  | -qBT +aAPC | 16.44 | 17.35 | 20.27 | 44.02 | 50.77 | 28.27 | 29.52 | 14.62 |
|  | +qBT -aAPC | 66.13 | 76.72 | 80.50 | 67.15 | 68.18 | 65.35 | 70.67 | 6.34 |
|  | +qBT +aAPC | 69.09 | 77.24 | 82.89 | 63.84 | 58.31 | 57.74 | 68.19 | 10.24 |
|  |  |  |  |  |  |  |  |  |  |
| **Figure 1E** | % T_SCM_ of CD8 T cells | Donor S9 | Donor S10 | Donor S11 | Donor S12 | Donor S13 | Donor S14 | Mean | SD |
|  | -qBT -aAPC | 66.64 | 73.82 | 82.31 | 65.72 | 65.59 | 64.66 | 69.79 | 6.98 |
|  | -qBT +aAPC | 10.57 | 22.76 | 26.87 | 60.01 | 54.93 | 40.97 | 36.02 | 19.31 |
|  | +qBT -aAPC | 84.88 | 86.92 | 89.13 | 75.84 | 68.17 | 70.75 | 79.28 | 8.89 |
|  | +qBT +aAPC | 84.19 | 82.65 | 81.72 | 67.17 | 59.52 | 64.69 | 73.32 | 10.76 |

| **Figure 1H** | Total flux (photons/sec) | Vehicle | | | | Pan-T (qBT) | | | | CAR-T (qBT) | | | | | | | | CAR-T (qBT+aAPC) | | | | | | | |
| --- | --- | --- | --- | --- | --- | --- | --- | --- | --- | --- | --- | --- | --- | --- | --- | --- | --- | --- | --- | --- | --- | --- | --- | --- | --- |
| **Day** | -1 | 1.12E+07 | 1.09E+07 | 1.06E+07 | 9.87E+06 | 1.12E+07 | 1.07E+07 | 1.04E+07 | 1.02E+07 | 1.12E+07 | 1.07E+07 | 1.07E+07 | 1.06E+07 | 1.05E+07 | 1.03E+07 | 1.03E+07 | 1.01E+07 | 1.11E+07 | 1.08E+07 | 1.07E+07 | 1.06E+07 | 1.05E+07 | 1.03E+07 | 1.02E+07 | 1.01E+07 |
|  | 6 | 9.84E+07 | 1.48E+08 | 1.20E+08 | 5.26E+07 | 1.86E+08 | 1.27E+08 | 1.09E+08 | 5.18E+07 | 3.96E+07 | 2.40E+07 | 2.72E+07 | 2.79E+07 | 2.14E+07 | 4.97E+07 | 3.38E+07 | 1.71E+07 | 1.46E+07 | 2.73E+07 | 1.64E+07 | 5.20E+07 | 1.69E+07 | 5.77E+07 | 1.97E+07 | 1.23E+07 |
|  | 10 | 3.42E+08 | 4.99E+08 | 2.96E+08 | 1.95E+08 | 4.64E+08 | 3.35E+08 | 1.47E+08 | 1.22E+08 | 2.04E+07 | 4.97E+07 | 1.36E+07 | 2.19E+07 | 2.33E+07 | 1.63E+07 | 1.87E+07 | 1.20E+07 | 2.52E+07 | 1.74E+07 | 1.42E+07 | 2.46E+07 | 1.26E+07 | 4.13E+07 | 1.73E+07 | 1.22E+07 |
|  | 14 |  | 1.79E+09 | 1.72E+09 | 2.99E+07 | 1.65E+09 | 1.13E+09 | 5.64E+08 | 3.69E+08 | 5.20E+06 | 4.97E+07 | 2.32E+06 | 5.04E+06 | 2.30E+07 | 4.77E+06 | 4.32E+06 | 4.87E+06 | 3.82E+07 | 5.12E+06 | 2.68E+06 | 3.03E+06 | 3.01E+06 | 7.55E+06 | 5.01E+06 | 5.43E+06 |
|  | 21 |  |  |  |  | 6.75E+06 | 3.71E+07 | 1.45E+07 | 2.70E+07 | 3.43E+07 | 5.03E+06 | 2.63E+06 | 2.33E+06 | 2.69E+06 | 2.48E+06 | 2.77E+06 | 2.22E+06 | 2.16E+07 | 2.67E+06 | 2.58E+06 | 3.03E+06 | 2.37E+06 | 2.60E+06 | 2.42E+06 | 5.12E+06 |
|  | 28 |  |  |  |  | 5.65E+06 | 1.38E+07 | 4.86E+06 | 4.50E+06 | 1.65E+07 | 4.66E+06 | 4.52E+06 | 4.14E+06 | 4.65E+06 | 7.12E+06 | 4.69E+06 | 4.28E+06 | 5.96E+06 | 4.48E+06 | 4.49E+06 | 1.33E+07 | 4.62E+06 | 4.66E+06 | 4.58E+06 | 4.34E+06 |
|  | 34 |  |  |  |  | 4.23E+06 | 1.13E+07 | 4.05E+06 | 3.79E+06 | 4.20E+06 | 3.89E+06 | 4.01E+06 | 3.79E+06 | 4.15E+06 | 4.65E+06 | 4.16E+06 | 3.89E+06 | 4.46E+06 | 4.39E+06 | 4.59E+06 | 1.72E+07 | 4.16E+06 | 4.11E+06 | 4.15E+06 | 4.01E+06 |
|  | 41 |  |  |  |  | 6.58E+06 | 3.94E+07 | 6.33E+06 | 6.02E+06 | 5.82E+06 | 5.83E+06 | 5.81E+06 | 5.65E+06 | 5.82E+06 | 6.80E+06 | 5.68E+06 | 5.57E+06 | 6.16E+06 | 6.18E+06 | 7.12E+06 | 1.91E+08 | 5.95E+06 | 5.87E+06 | 5.86E+06 | 5.69E+06 |
|  | 48 |  |  |  |  | 3.78E+07 | 2.18E+08 | 4.27E+07 | 2.18E+07 | 1.92E+07 | 1.97E+07 | 2.11E+07 | 1.88E+07 | 2.05E+07 | 3.22E+07 | 2.06E+07 | 1.91E+07 | 3.52E+07 | 5.00E+07 | 3.62E+07 | 1.72E+09 | 2.04E+07 | 2.06E+07 | 2.08E+07 | 1.96E+07 |
|  | 55 |  |  |  |  | 3.15E+06 |  | 3.29E+06 | 4.05E+06 | 1.42E+07 | 1.43E+07 | 2.82E+07 | 1.38E+07 | 1.33E+07 | 6.12E+07 | 1.34E+07 | 1.27E+07 | 1.60E+07 | 1.34E+07 | 1.39E+07 |  | 3.32E+07 | 1.49E+07 | 1.54E+07 | 1.38E+07 |
|  | 62 |  |  |  |  | 5.01E+06 |  | 1.22E+07 | 5.67E+07 | 3.67E+06 | 3.55E+06 | 3.58E+06 | 3.43E+06 | 3.37E+06 | 9.41E+06 | 3.47E+06 | 3.28E+06 | 2.19E+07 | 3.35E+06 | 3.34E+06 |  | 9.79E+07 | 3.52E+06 | 3.37E+06 | 3.36E+06 |
|  | 69 |  |  |  |  | 9.96E+06 |  | 1.12E+08 | 6.05E+08 | 8.80E+06 | 8.68E+06 | 8.89E+06 | 8.46E+06 | 9.31E+06 | 1.94E+07 | 9.41E+06 | 9.01E+06 | 1.26E+08 | 9.67E+06 | 9.26E+06 |  | 1.07E+09 | 1.44E+07 | 1.10E+07 | 9.99E+06 |
|  | 76 |  |  |  |  | 1.71E+07 |  | 8.30E+07 | 3.98E+09 | 9.90E+06 | 9.87E+06 | 9.92E+06 | 9.58E+06 | 1.00E+07 | 3.21E+07 | 1.01E+07 | 9.94E+06 | 7.32E+08 | 1.37E+07 | 1.11E+07 |  | 3.03E+08 | 1.17E+07 | 1.06E+07 | 1.00E+07 |
|  | 83 |  |  |  |  | 1.54E+07 |  | 2.23E+09 |  | 1.00E+07 | 1.00E+07 | 1.01E+07 | 1.00E+07 | 1.06E+07 | 4.21E+07 | 1.07E+07 | 1.03E+07 | 8.45E+08 | 1.57E+07 | 1.18E+07 |  | 3.76E+07 | 1.07E+07 | 1.05E+07 | 1.03E+07 |

| **Figure 2A** | %CAR^+^ | Donor 1 | Donor 2 | Donor 3 | Donor 4 | Donor 5 | Donor 6 | Donor 7 | Donor 8 | Donor 9 | Mean | SD |
| --- | --- | --- | --- | --- | --- | --- | --- | --- | --- | --- | --- | --- |
|  | Day 1 | 29.71 | 35.33 | 16.57 | 23.15 | 21.93 | 15.32 | 14.34 | 25.70 | 11.39 | 21.49 | 7.87 |
|  | Day 8 | 61.17 | 63.30 | 50.79 | 43.46 | 51.12 | 59.60 | 56.69 | 65.43 | 49.70 | 55.70 | 7.33 |
|  | Day 10 | 61.41 | 58.80 | 44.27 | 47.61 | 52.16 | 56.55 | 62.74 | 71.40 | 55.82 | 56.75 | 8.20 |
|  |  |  |  |  |  |  |  |  |  |  |  |  |
| **Figure 2B** | % qPBase^+^ | Donor 1 | Donor 2 | Donor 3 | Donor 4 | Donor 5 | Donor 6 | Donor 7 | Donor 8 | Donor 9 | Mean | SD |
|  | Day 1 | 63.34 | 68.10 | 38.35 | 41.66 | 39.45 | 34.79 | 38.57 | 65.79 | 35.75 | 47.31 | 14.02 |
|  | Day 8 | 0.23 | 0.51 | 0.04 | 0.08 | 0.08 | 0.07 | 0.06 | 0.28 | 0.06 | 0.16 | 0.16 |
|  | Day 10 | 0.06 | 0.14 | 0.01 | 0.11 | 0.08 | 0.01 | 0.09 | 0.09 | 0.11 | 0.08 | 0.04 |
|  |  |  |  |  |  |  |  |  |  |  |  |  |
| **Figure 2C** | Fold change | Donor 1 | Donor 2 | Donor 3 | Donor 4 | Donor 5 | Donor 6 | Donor 7 | Donor 8 | Donor 9 | Mean | SD |
|  |  | 162.24 | 146.03 | 162.85 | 256.21 | 204.11 | 120.87 | 162.57 | 206.01 | 188.89 | 178.86 | 39.84 |
|  |  |  |  |  |  |  |  |  |  |  |  |  |
| **Figure 2D** | %CAR^+^ cells | Donor 1 | Donor 2 | Donor 3 | Donor 4 | Donor 5 | Donor 6 | Donor 7 | Donor 8 | Donor 9 | Mean | SD |
|  | CD4 | 48.89 | 29.84 | 16.08 | 19.38 | 25.70 | 10.62 | 12.76 | 24.79 | 9.79 | 21.98 | 12.31 |
|  | CD8 | 46.14 | 65.30 | 78.68 | 75.37 | 69.22 | 85.91 | 84.70 | 71.44 | 85.50 | 73.58 | 12.71 |
|  |  |  |  |  |  |  |  |  |  |  |  |  |
| **Figure 2E** | % of CD4^+^ CAR^+^ Cells | Donor 1 | Donor 2 | Donor 3 | Donor 4 | Donor 5 | Donor 6 | Donor 7 | Donor 8 | Donor 9 | Mean | SD |
|  | T_N_ | 6.73 | 5.27 | 16.43 | 19.12 | 19.59 | 9.09 | 3.74 | 7.82 | 3.40 | 10.13 | 6.50 |
|  | T_SCM_ | 66.30 | 58.33 | 52.39 | 65.75 | 74.12 | 77.07 | 70.28 | 78.67 | 77.54 | 68.94 | 9.13 |
|  | T_CM_ | 20.99 | 27.67 | 25.70 | 11.81 | 4.67 | 8.85 | 16.01 | 9.27 | 8.76 | 14.86 | 8.20 |
|  | T_EM_ | 1.89 | 3.30 | 3.51 | 1.46 | 0.46 | 2.12 | 3.46 | 1.35 | 2.87 | 2.27 | 1.08 |
|  | T_EFF_ | 4.09 | 5.44 | 1.97 | 1.86 | 1.16 | 2.87 | 6.51 | 2.89 | 7.42 | 3.80 | 2.21 |
|  |  |  |  |  |  |  |  |  |  |  |  |  |
|  | % of CD8^+^ CAR^+^ Cells | Donor 1 | Donor 2 | Donor 3 | Donor 4 | Donor 5 | Donor 6 | Donor 7 | Donor 8 | Donor 9 | Mean | SD |
|  | T_N_ | 10.11 | 8.57 | 25.38 | 22.45 | 23.69 | 14.37 | 1.96 | 13.50 | 2.35 | 13.60 | 8.80 |
|  | T_SCM_ | 79.27 | 78.89 | 67.27 | 72.97 | 73.41 | 81.77 | 88.81 | 82.17 | 93.96 | 79.84 | 8.19 |
|  | T_CM_ | 6.06 | 6.62 | 5.91 | 1.31 | 0.41 | 2.62 | 1.79 | 1.04 | 0.61 | 2.93 | 2.54 |
|  | T_EM_ | 0.66 | 0.80 | 0.29 | 0.58 | 0.39 | 0.09 | 0.28 | 0.17 | 0.16 | 0.38 | 0.25 |
|  | T_EFF_ | 3.90 | 5.12 | 1.15 | 2.69 | 2.10 | 1.15 | 7.15 | 3.12 | 2.92 | 3.26 | 1.93 |
|  |  |  |  |  |  |  |  |  |  |  |  |  |
| **Figure 2F** | % | Donor 1 | Donor 2 | Donor 3 | Donor 4 | Donor 5 | Donor 6 | Donor 7 | Donor 8 | Donor 9 | Mean | SD |
|  | PD-1 | 0.85 | 1.36 | 0.92 | 0.31 | 2.15 | 0.55 | 0.77 | 0.77 | 0.46 | 0.90 | 0.56 |
|  | TIM-3 | 0.31 | 0.70 | 1.43 | 0.02 | 0.27 | 2.75 | 0.23 | 0.61 | 0.07 | 0.71 | 0.88 |
|  | LAG-3 | 1.13 | 1.30 | 2.25 | 4.16 | 3.03 | 3.52 | 1.11 | 0.55 | 3.11 | 2.24 | 1.27 |
|  |  |  |  |  |  |  |  |  |  |  |  |  |
| **Figure 2G** | % | Donor 1 | Donor 2 | Donor 3 | Donor 4 | Donor 5 | Donor 6 | Donor 7 | Donor 8 | Donor 9 | Mean | SD |
|  | KLRG-1 | 8.44 | 14.73 | 5.24 | 2.74 | 16.43 | 1.38 | 18.61 | 8.93 | 12.78 | 9.92 | 6.11 |
|  | CD57 | 0.19 | 0.44 | 0.56 | 0.05 | 0.13 | 0.20 | 0.19 | 0.35 | 0.05 | 0.24 | 0.18 |

| **Figure 3A** | % Specific Lysis | E:T=5:1 | | | | | E:T=1:1 | | | | | E:T=1:5 | | | | |
| --- | --- | --- | --- | --- | --- | --- | --- | --- | --- | --- | --- | --- | --- | --- | --- | --- |
|  |  | n1 | n2 | n3 | Mean | SD | n1 | n2 | n3 | Mean | SD | n1 | n2 | n3 | Mean | SD |
| Hours | 24 | 85.31 | 82.01 | 84.33 | 83.88 | 1.70 | 53.88 | 53.26 | 52.36 | 53.16 | 0.77 | 24.58 | 18.11 | 19.33 | 20.67 | 3.44 |
|  | 48 | 98.70 | 99.21 | 99.10 | 99.00 | 0.27 | 95.65 | 96.73 | 96.40 | 96.26 | 0.56 | 69.20 | 64.00 | 63.24 | 65.48 | 3.24 |
|  | 72 | 99.82 | 99.85 | 99.78 | 99.82 | 0.04 | 98.98 | 99.77 | 99.49 | 99.42 | 0.40 | 92.47 | 91.64 | 89.94 | 91.35 | 1.29 |
|  | 96 | 99.99 | NA | 99.98 | 99.99 | 0.01 | 99.99 | 99.98 | 99.98 | 99.98 | 0.01 | 99.81 | 99.63 | 99.29 | 99.58 | 0.26 |
|  |  |  |  |  |  |  |  |  |  |  |  |  |  |  |  |  |
| **Figure 3B** | % Specific Lysis | E:T=5:1 | | | | | E:T=1:1 | | | | | E:T=1:5 | | | | |
|  |  | n1 | n2 | n3 | Mean | SD | n1 | n2 | n3 | Mean | SD | n1 | n2 | n3 | Mean | SD |
| Hours | 24 | 57.28 | 70.82 | 69.47 | 65.85 | 7.46 | 21.71 | 36.05 | 27.96 | 28.57 | 7.19 | NA | 3.42 | -1.87 | 0.78 | 3.74 |
|  | 48 | 98.17 | 98.91 | 99.97 | 99.02 | 0.90 | 78.51 | 79.74 | 85.16 | 81.13 | 3.54 | NA | 14.54 | 13.81 | 14.17 | 0.51 |
|  | 72 | 99.77 | 99.78 | 100.00 | 99.85 | 0.13 | 97.41 | 96.59 | 98.56 | 97.52 | 0.99 | 40.52 | 45.68 | 44.66 | 43.62 | 2.73 |
|  | 96 | 100.00 | 99.99 | 100.00 | 100.00 | 0.00 | 99.94 | 99.86 | 99.93 | 99.91 | 0.04 | 99.14 | 88.20 | 89.64 | 92.32 | 5.94 |
|  |  |  |  |  |  |  |  |  |  |  |  |  |  |  |  |  |
| **Figure 3C** | IFN-γ | Pan-T | | | | | CAR-T | | | | |  |  |  |  |  |
|  |  | n1 | n2 | n3 | Mean | SD | n1 | n2 | n3 | Mean | SD |  |  |  |  |  |
|  | Donor 2 | 0.00 | 0.00 | 0.00 | 0.00 | 0.00 | 83.71 | 158.00 | 149.43 | 130.38 | 40.64 |  |  |  |  |  |
|  | Donor 1 | 0.00 | 0.00 | 0.00 | 0.00 | 0.00 | 186.57 | 220.86 | 180.86 | 196.10 | 21.63 |  |  |  |  |  |
|  |  |  |  |  |  |  |  |  |  |  |  |  |  |  |  |  |
| **Figure 3D** | TNF-α | Pan-T | | | | | CAR-T | | | | |  |  |  |  |  |
|  |  | n1 | n2 | n3 | Mean | SD | n1 | n2 | n3 | Mean | SD |  |  |  |  |  |
|  | Donor 2 | 3.66 | 3.66 | 3.66 | 3.66 | 0.00 | 392.90 | 261.72 | 388.60 | 347.74 | 74.53 |  |  |  |  |  |
|  | Donor 1 | 3.66 | 0.00 | 25.16 | 9.61 | 13.59 | 388.60 | 631.61 | NA | 510.11 | 171.83 |  |  |  |  |  |
|  |  |  |  |  |  |  |  |  |  |  |  |  |  |  |  |  |
| **Figure 3E** | IL-2 | Pan-T | | | | | CAR-T | | | | |  |  |  |  |  |
|  |  | n1 | n2 | n3 | Mean | SD | n1 | n2 | n3 | Mean | SD |  |  |  |  |  |
|  | Donor 2 | 0.00 | 0.00 | 0.00 | 0.00 | 0.00 | 2945.83 | 3287.50 | 3595.83 | 3276.39 | 325.14 |  |  |  |  |  |
|  | Donor 1 | 0.00 | 0.00 | 0.00 | 0.00 | 0.00 | 5845.83 | 6154.17 | 6462.50 | 6154.17 | 308.33 |  |  |  |  |  |

| **Figure 4C** | Total flux (photons/sec) | Vehicle | | | |  |  |  |  |  |  |  |  |
| --- | --- | --- | --- | --- | --- | --- | --- | --- | --- | --- | --- | --- | --- |
| **Day** | -1 | 2.18E+06 | 1.59E+06 | 1.56E+06 | 1.51E+06 |  |  |  |  |  |  |  |  |
|  | 6 | 1.72E+08 | 6.21E+07 | 8.36E+07 | 3.62E+07 |  |  |  |  |  |  |  |  |
|  | 9 | 4.87E+08 | 2.53E+08 | 1.81E+08 | 1.09E+08 |  |  |  |  |  |  |  |  |
|  | 13 | 5.93E+08 | 6.24E+08 | 1.34E+09 | 2.84E+08 |  |  |  |  |  |  |  |  |
|  | 21 |  |  |  |  |  |  |  |  |  |  |  |  |
|  | 28 |  |  |  |  |  |  |  |  |  |  |  |  |
|  | 34 |  |  |  |  |  |  |  |  |  |  |  |  |
|  | 41 |  |  |  |  |  |  |  |  |  |  |  |  |
|  | 49 |  |  |  |  |  |  |  |  |  |  |  |  |
|  | 56 |  |  |  |  |  |  |  |  |  |  |  |  |
|  | 62 |  |  |  |  |  |  |  |  |  |  |  |  |
|  | 70 |  |  |  |  |  |  |  |  |  |  |  |  |
|  | 77 |  |  |  |  |  |  |  |  |  |  |  |  |
|  | 84 |  |  |  |  |  |  |  |  |  |  |  |  |
|  | 91 |  |  |  |  |  |  |  |  |  |  |  |  |
|  | Total flux (photons/sec) | Pan-T (L) | | | | CAR-T (L) | | | | | | | |
| **Day** | -1 | 2.59E+06 | 1.50E+06 | 1.41E+06 | 1.40E+06 | 2.30E+06 | 1.96E+06 | 1.85E+06 | 1.62E+06 | 1.52E+06 | 1.51E+06 | 1.46E+06 | 1.42E+06 |
|  | 6 | 1.21E+07 | 1.96E+07 | 1.00E+08 | 2.47E+07 | 4.65E+06 | 5.14E+06 | 1.32E+07 | 1.64E+07 | 1.57E+07 | 1.43E+07 | 2.05E+07 | 6.83E+06 |
|  | 9 | 4.40E+07 | 8.24E+07 | 3.68E+08 | 6.76E+07 | 2.28E+06 | 7.18E+06 | 3.44E+06 | 9.16E+06 | 9.63E+06 | 3.60E+06 | 7.48E+06 | 4.46E+06 |
|  | 13 | 8.21E+07 | 1.37E+08 | 6.76E+08 | 8.58E+07 | 1.60E+06 | 1.90E+06 | 1.78E+06 | 2.45E+06 | 2.77E+06 | 2.07E+06 | 2.02E+06 | 2.02E+06 |
|  | 21 | 2.34E+07 | 3.97E+06 | 4.32E+07 | 4.01E+06 | 1.96E+06 | 1.99E+06 | 2.13E+06 | 2.12E+06 | 2.11E+06 | 2.00E+06 | 2.01E+06 | 1.76E+06 |
|  | 28 | 5.39E+06 | 3.70E+06 | 3.88E+06 | 3.17E+06 | 2.61E+06 | 2.45E+06 | 2.58E+06 | 2.22E+06 | 2.05E+06 | 2.20E+06 | 2.21E+06 | 1.94E+06 |
|  | 34 | 3.13E+06 | 2.74E+06 | 3.02E+06 | 2.60E+06 | 2.22E+06 | 2.19E+06 | 2.13E+06 | 2.05E+06 | 2.10E+06 | 2.13E+06 | 2.09E+06 | 1.89E+06 |
|  | 41 | 2.81E+06 | 2.80E+06 | 3.14E+06 | 2.70E+06 | 2.44E+06 | 2.59E+06 | 2.41E+06 | 2.25E+06 | 2.19E+06 | 2.25E+06 | 2.24E+06 | 2.01E+06 |
|  | 49 | 5.86E+06 | 3.49E+06 | 3.47E+06 | 3.14E+06 | 1.39E+07 | 3.54E+07 | 3.82E+06 | 2.77E+06 | 2.92E+06 | 2.93E+06 | 2.98E+06 | 2.76E+06 |
|  | 56 | 7.43E+06 | 3.38E+06 |  | 3.19E+06 |  | 8.94E+07 | 2.12E+07 | 3.03E+06 | 2.67E+06 | 2.70E+06 | 2.69E+06 | 2.53E+06 |
|  | 62 | 1.60E+07 | 3.14E+06 |  | 2.88E+06 |  | 1.85E+08 | 3.62E+07 | 2.47E+06 | 2.28E+06 | 2.40E+06 | 2.29E+06 | 2.25E+06 |
|  | 70 | 3.99E+08 | 4.13E+06 |  | 2.57E+06 |  | 2.53E+08 | 1.22E+08 | 2.54E+06 | 1.59E+06 | 1.60E+06 | 1.64E+06 | 1.59E+06 |
|  | 77 |  | 2.58E+06 |  | 2.52E+06 |  | 1.02E+08 | 9.76E+08 | 5.81E+06 | 1.64E+06 | 1.66E+06 | 1.64E+06 | 1.55E+06 |
|  | 84 |  | 2.01E+06 |  | 2.08E+06 |  | 3.71E+07 |  | 6.28E+06 | 1.47E+06 | 1.49E+06 | 1.56E+06 | 1.36E+06 |
|  | 91 |  | 2.74E+06 |  | 2.62E+06 |  | 4.09E+07 |  | 2.12E+06 | 1.88E+06 | 1.95E+06 | 2.01E+06 | 1.91E+06 |
|  | Total flux (photons/sec) | Pan-T (M) | | | | CAR-T (M) | | | | | | | |
| **Day** | -1 | 1.99E+06 | 1.87E+06 | 1.52E+06 | 1.50E+06 | 2.31E+06 | 1.98E+06 | 1.64E+06 | 1.58E+06 | 1.54E+06 | 1.53E+06 | 1.52E+06 | 1.46E+06 |
|  | 6 | 2.02E+07 | 6.60E+07 | 4.16E+07 | 3.60E+07 | 6.05E+06 | 8.58E+06 | 1.01E+07 | 5.34E+06 | 3.52E+06 | 3.76E+06 | 2.89E+06 | 8.16E+06 |
|  | 9 | 5.09E+07 | 1.33E+08 | 7.36E+07 | 1.35E+08 | 2.21E+06 | 2.89E+06 | 2.54E+06 | 2.09E+06 | 1.81E+06 | 1.79E+06 | 1.87E+06 | 1.85E+06 |
|  | 13 | 5.42E+06 | 1.81E+07 | 3.82E+07 | 6.63E+07 | 2.21E+06 | 2.24E+06 | 2.04E+06 | 1.94E+06 | 1.72E+06 | 1.96E+06 | 1.68E+07 | 1.85E+06 |
|  | 21 | 1.72E+06 | 1.82E+06 | 2.01E+06 | 1.95E+06 | 1.55E+06 | 1.62E+06 | 1.66E+06 | 1.40E+06 | 1.70E+06 | 1.82E+06 | 1.77E+06 | 1.58E+06 |
|  | 28 | 2.17E+06 | 2.19E+06 | 2.29E+06 | 2.01E+06 | 2.22E+06 | 2.24E+06 | 2.34E+06 | 2.05E+06 | 2.20E+06 | 2.35E+06 | 2.41E+06 | 2.13E+06 |
|  | 34 | 1.79E+06 | 1.79E+06 | 1.83E+06 | 1.62E+06 | 1.83E+06 | 1.75E+06 | 1.81E+06 | 1.65E+06 | 1.70E+06 | 1.66E+06 | 1.65E+06 | 1.56E+06 |
|  | 41 | 1.94E+06 | 1.97E+06 | 2.14E+06 | 1.92E+06 | 1.96E+06 | 1.97E+06 | 2.01E+06 | 1.89E+06 | 2.05E+06 | 2.00E+06 | 2.00E+06 | 1.89E+06 |
|  | 49 | 2.64E+06 | 2.63E+06 | 2.78E+06 | 2.50E+06 | 2.54E+06 | 2.50E+06 | 2.63E+06 | 2.38E+06 | 2.47E+06 | 2.47E+06 | 2.56E+06 | 2.38E+06 |
|  | 56 | 2.38E+06 | 2.42E+06 | 2.36E+06 |  | 2.27E+06 | 2.24E+06 | 2.25E+06 | 2.08E+06 | 2.25E+06 | 2.32E+06 | 2.36E+06 | 2.14E+06 |
|  | 62 | 1.93E+06 | 1.96E+06 |  |  | 2.11E+06 | 2.09E+06 | 2.12E+06 |  | 2.03E+06 | 1.99E+06 | 1.97E+06 | 1.86E+06 |
|  | 70 | 1.62E+06 | 1.65E+06 |  |  | 1.65E+06 | 1.66E+06 | 1.71E+06 |  | 1.62E+06 | 1.71E+06 | 1.63E+06 | 1.60E+06 |
|  | 77 |  | 1.37E+06 |  |  | 1.29E+06 | 1.36E+06 |  |  | 1.34E+06 | 1.34E+06 | 1.32E+06 | 1.27E+06 |
|  | 84 |  | 1.21E+06 |  |  | 1.18E+06 | 1.16E+06 |  |  |  | 1.08E+06 | 1.08E+06 | 1.01E+06 |
|  | 91 |  | 1.76E+06 |  |  | 1.52E+06 | 1.53E+06 |  |  |  | 1.57E+06 | 1.46E+06 |  |
|  | Total flux (photons/sec) | Pan-T (H) | | | | CAR-T (H) | | | | | | | |
| **Day** | -1 | 2.22E+06 | 1.81E+06 | 1.50E+06 | 1.37E+06 | 2.22E+06 | 1.91E+06 | 1.84E+06 | 1.76E+06 | 1.56E+06 | 1.51E+06 | 1.50E+06 | 1.40E+06 |
|  | 6 | 1.29E+07 | 7.70E+07 | 1.90E+07 | 1.17E+07 | 2.54E+06 | 2.46E+06 | 2.54E+06 | 2.63E+06 | 2.47E+06 | 2.54E+06 | 2.51E+06 | 2.56E+06 |
|  | 9 | 1.04E+07 | 3.05E+07 | 3.91E+07 | 7.98E+06 | 1.74E+06 | 1.87E+06 | 1.88E+06 | 1.71E+06 | 1.73E+06 | 1.81E+06 | 1.83E+06 | 1.73E+06 |
|  | 13 | 2.64E+06 | 3.13E+06 | 3.96E+06 | 1.87E+06 | 1.65E+06 | 1.73E+06 | 1.86E+06 | 2.03E+06 | 2.21E+06 | 2.24E+06 | 2.07E+06 | 1.98E+06 |
|  | 21 | 1.78E+06 | 1.75E+06 | 1.92E+06 | 1.69E+06 | 1.84E+06 | 1.88E+06 | 1.95E+06 | 1.76E+06 | 1.88E+06 | 2.01E+06 | 1.95E+06 | 1.73E+06 |
|  | 28 | 2.22E+06 | 2.37E+06 | 2.47E+06 | 2.14E+06 | 5.78E+06 | 5.82E+06 |  | 5.76E+06 | 2.31E+06 | 2.33E+06 | 2.48E+06 | 2.18E+06 |
|  | 34 |  | 1.89E+06 | 1.87E+06 | 1.69E+06 | 1.83E+06 | 1.88E+06 |  | 1.71E+06 | 1.92E+06 | 1.87E+06 | 1.87E+06 | 1.68E+06 |
|  | 41 |  | 1.89E+06 |  |  | 1.97E+06 | 2.00E+06 |  | 1.85E+06 | 1.91E+06 | 1.91E+06 | 1.98E+06 | 1.90E+06 |
|  | 49 |  | 2.61E+06 |  |  | 2.57E+06 | 2.69E+06 |  | 2.57E+06 | 2.66E+06 |  | 2.60E+06 |  |
|  | 56 |  |  |  |  | 2.17E+06 | 2.12E+06 |  | 2.14E+06 | 2.12E+06 |  | 2.16E+06 |  |
|  | 62 |  |  |  |  | 1.99E+06 | 2.02E+06 |  | 1.89E+06 |  |  | 2.00E+06 |  |
|  | 70 |  |  |  |  | 1.66E+06 | 1.55E+06 |  | 1.52E+06 |  |  | 1.55E+06 |  |
|  | 77 |  |  |  |  | 1.43E+06 | 1.48E+06 |  | 1.40E+06 |  |  | 1.34E+06 |  |
|  | 84 |  |  |  |  | 1.06E+06 | 1.09E+06 |  | 1.03E+06 |  |  | 1.01E+06 |  |
|  | 91 |  |  |  |  | 1.49E+06 |  |  |  |  |  | 1.33E+06 |  |
|  |  |  |  |  |  | Rechallenge |  |  |  |  |  |  |  |

| **Figure 5E** | Human CD45^+^ T cells in blood (cells/mL) | CtrlT | | | | CARN87 (Lentiviral) | | | |  |  |  | |
| --- | --- | --- | --- | --- | --- | --- | --- | --- | --- | --- | --- | --- | --- |
| Lentiviral | Day 11 | 1.07E+03 | 1.14E+03 | 9.45E+02 | 1.40E+03 | 5.80E+02 | 8.60E+03 | 1.34E+03 | 1.47E+03 |  |  |  |  |
|  | Day 48 | 1.00E+00 | 1.00E+00 | 1.13E+02 | 4.45E+01 | 1.61E+02 | 2.54E+04 | 8.51E+02 | 1.38E+02 |  |  |  |  |
|  | Human CD45^+^ T cells in blood (cells/mL) | CARN87 (qPB) | | | | PanT | | | CARiC9-20/19 | | | | |
| qPB | Day 11 | 8.65E+04 | 1.19E+05 | 3.83E+05 | 1.62E+05 | 2.26E+03 | 5.00E+03 | 1.64E+03 | 8.26E+03 | 9.49E+03 | 2.44E+03 | 3.95E+03 |  |
|  | Day 48 | 1.25E+07 | 1.64E+06 | 4.47E+07 | 8.23E+07 | 1.35E+03 | 1.00E+00 | 9.08E+03 | 2.11E+06 | 4.49E+05 | 2.13E+04 | 1.59E+05 |  |

| **Figure 6A** | CD8/CD4 ratio | Donor 3 | Donor 4 | Donor 5 | Donor 6 | Donor 7 | Donor 9 | Mean | SD |  |  |
| --- | --- | --- | --- | --- | --- | --- | --- | --- | --- | --- | --- |
| Healthy | PBMC | 1.00 | 0.95 | 0.68 | 0.66 | 0.98 | 0.83 | 0.85 | 0.15 |  |  |
|  | CAR-T | 5.25 | 3.73 | 2.72 | 6.12 | 7.26 | 8.75 | 5.64 | 2.23 |  |  |
|  |  |  |  |  |  |  |  |  |  |  |  |
|  | CD8/CD4 ratio | DLBCL1 | DLBCL2 | DLBCL3 | CLL1 | CLL2 | CLL3 | HL | MM | Mean | SD |
| Patient | PBMC | 1.09 | 1.67 | 2.89 | 0.70 | 0.30 | 0.37 | 0.52 | 1.78 | 1.17 | 0.90 |
|  | CAR-T | 1.85 | 1.09 | 3.72 | 0.78 | 0.55 | 0.57 | 1.53 | 1.50 | 1.45 | 1.03 |
|  |  |  |  |  |  |  |  |  |  |  |  |
| **Figure 6B** | % CD4^+^ Cells | DLBCL1 | DLBCL2 | DLBCL3 | CLL1 | CLL2 | CLL3 | HL | MM | Mean | SD |
| Pre-nucleofection | T_N_ | 15.90 | 11.11 | 5.52 | 39.15 | 52.58 | 39.39 | 23.40 | 13.39 | 25.06 | 16.74 |
|  | T_SCM_ | 19.49 | 12.96 | 7.04 | 10.38 | 10.31 | 16.41 | 16.47 | 16.94 | 13.75 | 4.25 |
|  | T_CM_ | 20.43 | 50.67 | 44.40 | 19.04 | 10.95 | 30.75 | 36.90 | 23.85 | 29.62 | 13.60 |
|  | T_EM_ | 21.50 | 19.58 | 38.05 | 6.88 | 9.54 | 12.31 | 18.12 | 16.21 | 17.77 | 9.62 |
|  | T_EFF_ | 22.69 | 5.68 | 4.99 | 24.56 | 16.62 | 1.14 | 5.11 | 29.60 | 13.80 | 10.90 |
|  |  |  |  |  |  |  |  |  |  |  |  |
|  | % CD8^+^ Cells | DLBCL1 | DLBCL2 | DLBCL3 | CLL1 | CLL2 | CLL3 | HL | MM | Mean | SD |
| Pre-nucleofection | T_N_ | 21.39 | 10.05 | 15.86 | 32.37 | 36.78 | 40.28 | 26.20 | 8.70 | 23.95 | 11.98 |
|  | T_SCM_ | 8.15 | 6.19 | 10.59 | 5.67 | 5.53 | 4.48 | 15.36 | 13.20 | 8.65 | 4.00 |
|  | T_CM_ | 3.26 | 3.86 | 10.84 | 2.89 | 2.40 | 17.20 | 11.59 | 7.30 | 7.42 | 5.35 |
|  | T_EM_ | 7.04 | 8.94 | 19.01 | 3.45 | 3.85 | 19.32 | 13.85 | 3.00 | 9.81 | 6.78 |
|  | T_EFF_ | 60.16 | 70.97 | 43.70 | 55.62 | 51.44 | 18.73 | 33.00 | 67.80 | 50.18 | 17.73 |
|  |  |  |  |  |  |  |  |  |  |  |  |
|  | % of CD4^+^ CAR^+^ Cells | DLBCL1 | DLBCL2 | DLBCL3 | CLL1 | CLL2 | CLL3 | HL | MM | Mean | SD |
| Post-nucleofection | T_N_ | 11.96 | 11.70 | 0.66 | 11.55 | 6.38 | 0.70 | 8.97 | 17.15 | 8.63 | 5.77 |
|  | T_SCM_ | 66.26 | 66.36 | 77.55 | 80.25 | 83.87 | 88.04 | 77.18 | 70.47 | 76.25 | 7.99 |
|  | T_CM_ | 10.99 | 11.41 | 10.85 | 7.16 | 7.50 | 4.68 | 7.26 | 4.76 | 8.08 | 2.72 |
|  | T_EM_ | 2.95 | 3.05 | 4.95 | 0.29 | 0.69 | 1.16 | 2.90 | 2.86 | 2.36 | 1.54 |
|  | T_EFF_ | 7.84 | 7.48 | 5.99 | 0.75 | 1.55 | 5.41 | 3.69 | 4.76 | 4.68 | 2.57 |
|  |  |  |  |  |  |  |  |  |  |  |  |
|  | % of CD8^+^ CAR^+^ Cells | DLBCL1 | DLBCL2 | DLBCL3 | CLL1 | CLL2 | CLL3 | HL | MM | Mean | SD |
| Post-nucleofection | T_N_ | 16.53 | 15.33 | 0.33 | 12.84 | 4.84 | 0.45 | 11.83 | 21.97 | 10.52 | 7.88 |
|  | T_SCM_ | 72.55 | 74.81 | 88.95 | 83.76 | 90.22 | 95.43 | 80.48 | 68.79 | 81.87 | 9.39 |
|  | T_CM_ | 0.22 | 0.75 | 1.54 | 1.22 | 1.17 | 0.45 | 0.69 | 3.50 | 1.19 | 1.03 |
|  | T_EM_ | 0.36 | 0.21 | 1.25 | 0.20 | 0.23 | 0.09 | 0.52 | 0.00 | 0.36 | 0.39 |
|  | T_EFF_ | 10.34 | 8.90 | 7.93 | 1.98 | 3.54 | 3.58 | 6.48 | 5.73 | 6.06 | 2.91 |

| **Figure S1A** | % of CD4^+^CAR^+^ cells | n1 | n2 | n3 | n4 | n5 | n6 | Mean | SD |
| --- | --- | --- | --- | --- | --- | --- | --- | --- | --- |
| Conventional Plate (perfusion) | T_N_ | 3.97 | 4.66 | 6.95 | 11.22 | 14.06 | 9.37 | 8.37 | 3.92 |
|  | T_SCM_ | 90.35 | 82.68 | 69.22 | 78.69 | 71.87 | 65.10 | 76.32 | 9.37 |
|  | T_CM_ | 2.06 | 7.69 | 10.60 | 1.27 | 3.40 | 5.25 | 5.05 | 3.57 |
|  | T_EM_ | 0.19 | 2.02 | 7.60 | 2.38 | 4.85 | 8.05 | 4.18 | 3.19 |
|  | T_EFF_ | 3.43 | 2.95 | 5.63 | 6.45 | 5.81 | 12.24 | 6.09 | 3.32 |
|  |  |  |  |  |  |  |  |  |  |
|  | % of CD8+CAR+ cells | n1 | n2 | n3 | n4 | n5 | n6 | Mean | SD |
| Conventional Plate (perfusion) | T_N_ | 26.67 | 27.74 | 20.41 | 23.84 | 26.77 | 23.10 | 24.76 | 2.80 |
|  | T_SCM_ | 66.77 | 68.78 | 73.21 | 73.52 | 66.65 | 69.00 | 69.66 | 3.04 |
|  | T_CM_ | 0.52 | 0.47 | 0.55 | 0.05 | 0.40 | 0.46 | 0.41 | 0.18 |
|  | T_EM_ | 0.07 | 0.05 | 0.14 | 0.02 | 0.18 | 0.24 | 0.12 | 0.08 |
|  | T_EFF_ | 5.96 | 2.96 | 5.69 | 2.57 | 6.00 | 7.20 | 5.06 | 1.86 |
|  |  |  |  |  |  |  |  |  |  |
|  | % of CD4+CAR+ cells | n1 | n2 | n3 | n4 | n5 | n6 | Mean | SD |
| G-Rex (fed batch) | T_N_ | 17.72 | 15.54 | 10.87 | 25.06 | 24.59 | 8.15 | 16.99 | 6.94 |
|  | T_SCM_ | 77.25 | 72.72 | 84.55 | 50.73 | 59.13 | 53.23 | 66.27 | 13.85 |
|  | T_CM_ | 4.40 | 10.48 | 3.66 | 9.31 | 9.30 | 27.35 | 10.75 | 8.61 |
|  | T_EM_ | 0.16 | 0.47 | 0.33 | 7.84 | 2.69 | 6.89 | 3.06 | 3.47 |
|  | T_EFF_ | 0.47 | 0.79 | 0.59 | 7.07 | 4.28 | 4.38 | 2.93 | 2.73 |
|  |  |  |  |  |  |  |  |  |  |
|  | % of CD8+CAR+ cells | n1 | n2 | n3 | n4 | n5 | n6 | Mean | SD |
| G-Rex (fed batch) | T_N_ | 43.95 | 37.78 | 32.32 | 32.77 | 26.36 | 24.53 | 32.95 | 7.20 |
|  | T_SCM_ | 53.73 | 59.58 | 65.81 | 58.55 | 66.28 | 67.73 | 61.95 | 5.51 |
|  | T_CM_ | 1.12 | 1.76 | 0.89 | 2.06 | 2.18 | 3.76 | 1.96 | 1.02 |
|  | T_EM_ | 0.03 | 0.05 | 0.08 | 0.75 | 0.44 | 1.01 | 0.39 | 0.41 |
|  | T_EFF_ | 1.17 | 0.84 | 0.90 | 5.86 | 4.74 | 2.97 | 2.75 | 2.16 |
|  |  |  |  |  |  |  |  |  |  |
| **Figure S1B** | % of Lysis | Donor S1 | Donor S2 | Donor S3 | Donor S4 | Donor S5 | Donor S6 | Mean | SD |
| 48hr | Conventional plate | 17.95 | 17.42 | 1.57 | 22.94 | 43.82 | 5.95 | 18.28 | 14.87 |
|  | G-Rex | 55.45 | 70.98 | 22.55 | 83.95 | 65.01 | 67.29 | 60.87 | 20.93 |
|  |  |  |  |  |  |  |  |  |  |
|  | % of Lysis | Donor S1 | Donor S2 | Donor S3 | Donor S4 | Donor S5 | Donor S6 | Mean | SD |
| 72hr | Conventional plate | 15.58 | 31.48 | -4.12 | 35.46 | 42.04 | 10.72 | 21.86 | 17.45 |
|  | G-Rex | 75.47 | 97.86 | 37.84 | 98.99 | 88.16 | 95.41 | 82.29 | 23.46 |

| **Figure S2A** | CAR^+^ Cell Survival (%) | AP1903 | | | | | | | | | | | | | | | | | | | | | |
| --- | --- | --- | --- | --- | --- | --- | --- | --- | --- | --- | --- | --- | --- | --- | --- | --- | --- | --- | --- | --- | --- | --- | --- |
|  |  | 0 nM | | | | | 2.5nM | | | | | | 5nM | | | | | | 10nM | | | | |
|  |  | n1 | n2 | n3 | Mean | SD | n1 | n2 | n3 | Mean | SD | n1 | | n2 | n3 | Mean | SD | n1 | | n2 | n3 | Mean | SD |
|  | CD20CD19 CAR-T | 98.98 | 100.26 | 100.76 | 100.00 | 0.92 | 94.97 | 96.35 | 96.09 | 95.80 | 0.73 | 91.93 | | 94.01 | 93.13 | 93.02 | 1.04 | 92.66 | | 93.39 | 93.52 | 93.19 | 0.46 |
|  | GF-CART01 | 101.01 | 98.52 | 100.47 | 100.00 | 1.31 | 27.08 | 27.38 | 28.00 | 27.49 | 0.47 | 23.60 | | 22.03 | 22.60 | 22.74 | 0.79 | 19.51 | | 19.05 | 18.86 | 19.14 | 0.33 |

| **Figure S2B** | CAR Copy Number / Cell | Total Amount of DNA Transfected (mg) | | | | | | | | | | | |
| --- | --- | --- | --- | --- | --- | --- | --- | --- | --- | --- | --- | --- | --- |
|  |  | 5 | | 6 | | 7 | | 8 | | | 15 | | |
|  |  | 3.08 | 3.59 | 3.49 | 4.09 | 3.71 | 4.10 | 4.75 | 3.65 | 4.61 | 3.99 | 4.57 | 4.34 |

| **Figure S3** | % Specific Lysis (Raji-GFP/Luc) | E:T=5:1 | | | | | E:T=1:1 | | | | | E:T=1:5 | | | | |
| --- | --- | --- | --- | --- | --- | --- | --- | --- | --- | --- | --- | --- | --- | --- | --- | --- |
|  |  | n1 | n2 | n3 | Mean | SD | n1 | n2 | n3 | Mean | SD | n1 | n2 | n3 | Mean | SD |
| Hours | 24 | 8.36 | 6.61 | 6.39 | 7.12 | 1.08 | 3.64 | 5.11 | 5.81 | 4.85 | 1.11 | -1.47 | -0.74 | 4.16 | 0.65 | 3.06 |
|  | 48 | 49.70 | 46.46 | 50.54 | 48.90 | 2.15 | 17.76 | 17.53 | 15.50 | 16.93 | 1.24 | 5.38 | 0.78 | 4.81 | 3.66 | 2.51 |
|  | 72 | 96.44 | 94.81 | 94.93 | 95.39 | 0.91 | 56.36 | 53.98 | 48.14 | 52.83 | 4.23 | 22.71 | 18.60 | 17.34 | 19.55 | 2.81 |
|  | 96 | 99.67 | 99.69 | 99.68 | 99.68 | 0.01 | 78.83 | 75.27 | 71.69 | 75.26 | 3.57 | 36.26 | 31.16 | 28.41 | 31.94 | 3.98 |
|  |  |  |  |  |  |  |  |  |  |  |  |  |  |  |  |  |
|  | % Specific Lysis (Nalm6-GFP/Luc) | E:T=5:1 | | | | | E:T=1:1 | | | | | E:T=1:5 | | | | |
|  |  | n1 | n2 | n3 | Mean | SD | n1 | n2 | n3 | Mean | SD | n1 | n2 | n3 | Mean | SD |
| Hours | 24 | 6.17 | 3.93 | 6.37 | 5.49 | 1.35 | -10.03 | -8.98 | 6.02 | -4.33 | 8.98 | -8.53 | -5.25 | 13.44 | -0.11 | 11.85 |
|  | 48 | 66.32 | 60.93 | 54.91 | 60.72 | 5.71 | 19.14 | 24.16 | 39.53 | 27.61 | 10.62 | -2.04 | 5.87 | 3.27 | 2.37 | 4.03 |
|  | 72 | 92.27 | 92.85 | 90.82 | 91.98 | 1.05 | 51.02 | 57.97 | 73.06 | 60.68 | 11.27 | 19.49 | 13.02 | 23.99 | 18.83 | 5.51 |
|  | 96 | 94.91 | 94.68 | 94.69 | 94.76 | 0.13 | 71.22 | 78.06 | 87.38 | 78.89 | 8.11 | 12.65 | 25.24 | 29.37 | 22.42 | 8.71 |

| **Figure S4A** | IFN-γ (pg/ml) | CAR-T (L) | | | | | | CAR-T (M) | | | | | | CAR-T (H) | | | | | |
| --- | --- | --- | --- | --- | --- | --- | --- | --- | --- | --- | --- | --- | --- | --- | --- | --- | --- | --- | --- |
|  |  | n1 | n2 | n3 | n4 | Mean | SD | n1 | n2 | n3 | n4 | Mean | SD | n1 | n2 | n3 | n4 | Mean | SD |
| Days Post T cell Injection | 2 | 0.00 | 0.00 | 0.00 | 0.00 | 0.00 | 0.00 | 21.56 | 25.71 | 105.45 | 5.45 | 39.55 | 44.80 | 42.34 | 0.00 | 72.73 | 132.99 | 62.01 | 55.93 |
|  | 5 | NA | NA | NA | NA | NA | NA | 254.29 | 0.00 | 48.57 | 497.40 | 200.06 | 226.81 | 0.00 | 0.00 | 0.00 | 43.64 | 10.91 | 21.82 |
|  | 8 | 0.00 | 0.00 | 0.00 | 0.00 | 0.00 | 0.00 | 0.00 | 0.00 | 0.00 | 0.00 | 0.00 | 0.00 | 0.00 | 0.00 | 0.00 | 0.00 | 0.00 | 0.00 |
|  | 14 | 0.00 | 0.00 | 0.00 | 0.00 | 0.00 | 0.00 | 0.00 | 0.00 | 0.00 | 0.00 | 0.00 | 0.00 | 5.97 | 0.00 | 0.00 | 140.52 | 36.62 | 69.32 |
|  |  |  |  |  |  |  |  |  |  |  |  |  |  |  |  |  |  |  |  |
|  | TNF-α (pg/ml) | CAR-T (L) | | | | | | CAR-T (M) | | | | | | CAR-T (H) | | | | | |
|  |  | n1 | n2 | n3 | n4 | Mean | SD | n1 | n2 | n3 | n4 | Mean | SD | n1 | n2 | n3 | n4 | Mean | SD |
| **Days Post T cell Injection** | 2 | 0.00 | 0.00 | 0.00 | 0.00 | 0.00 | 0.00 | 0.00 | 0.00 | 0.00 | 0.00 | 0.00 | 0.00 | 0.00 | 0.00 | 0.00 | 0.00 | 0.00 | 0.00 |
|  | 5 | 0.00 | 0.00 | 0.00 | 0.00 | 0.00 | 0.00 | 0.00 | 0.00 | 0.00 | 0.00 | 0.00 | 0.00 | 0.00 | 0.00 | 0.00 | 0.00 | 0.00 | 0.00 |
|  | 8 | 0.00 | 0.00 | 0.00 | 0.00 | 0.00 | 0.00 | 0.00 | 0.00 | 0.00 | 0.00 | 0.00 | 0.00 | 0.00 | 0.00 | 0.00 | 0.00 | 0.00 | 0.00 |
|  | 14 | 0.00 | 0.00 | 0.00 | 0.00 | 0.00 | 0.00 | 0.00 | 0.00 | 0.00 | 0.00 | 0.00 | 0.00 | 0.00 | 0.00 | 0.00 | 0.00 | 0.00 | 0.00 |
|  |  |  |  |  |  |  |  |  |  |  |  |  |  |  |  |  |  |  |  |
|  | IL-2 (pg/ml) | CAR-T (L) | | | | | | CAR-T (M) | | | | | | CAR-T (H) | | | | | |
|  |  | n1 | n2 | n3 | n4 | Mean | SD | n1 | n2 | n3 | n4 | Mean | SD | n1 | n2 | n3 | n4 | Mean | SD |
| **Days Post T cell Injection** | 2 | 0.00 | 0.00 | 0.00 | 2.55 | 0.64 | 1.28 | 27.65 | 34.80 | 56.63 | 59.69 | 44.69 | 15.87 | 1.33 | 0.00 | 19.90 | 133.98 | 38.80 | 64.10 |
|  | 5 | 0.00 | 0.00 | 0.00 | 0.00 | 0.00 | 0.00 | 0.00 | 0.00 | 0.00 | 0.00 | 0.00 | 0.00 | 0.00 | 0.00 | 0.00 | 0.00 | 0.00 | 0.00 |
|  | 8 | 0.00 | 0.00 | 0.00 | 0.00 | 0.00 | 0.00 | 0.00 | 0.00 | 0.00 | 0.00 | 0.00 | 0.00 | 0.00 | 0.00 | 0.00 | 0.00 | 0.00 | 0.00 |
|  | 14 | 0.00 | 0.00 | 0.00 | 0.00 | 0.00 | 0.00 | 0.00 | 0.00 | 0.00 | 0.00 | 0.00 | 0.00 | 0.00 | 0.00 | 0.00 | 0.00 | 0.00 | 0.00 |
|  |  |  |  |  |  |  |  |  |  |  |  |  |  |  |  |  |  |  |  |
|  | IL-6 (pg/ml) | CAR-T (L) | | | | | | CAR-T (M) | | | | | | CAR-T (H) | | | | | |
|  |  | n1 | n2 | n3 | n4 | Mean | SD | n1 | n2 | n3 | n4 | Mean | SD | n1 | n2 | n3 | n4 | Mean | SD |
| **Days Post T cell Injection** | 2 | 0.00 | 0.00 | 0.00 | 0.00 | 0.00 | 0.00 | 0.00 | 0.00 | 0.00 | 0.00 | 0.00 | 0.00 | 0.00 | 0.00 | 0.00 | 0.00 | 0.00 | 0.00 |
|  | 5 | 0.00 | 0.00 | 0.00 | 0.00 | 0.00 | 0.00 | 0.00 | 0.00 | 0.00 | 0.00 | 0.00 | 0.00 | 0.00 | 0.00 | 0.00 | 0.00 | 0.00 | 0.00 |
|  | 8 | 0.00 | 0.00 | 0.00 | 0.00 | 0.00 | 0.00 | 0.00 | 0.00 | 0.00 | 0.00 | 0.00 | 0.00 | 0.00 | 0.00 | 0.00 | 0.00 | 0.00 | 0.00 |
|  | 14 | 0.00 | 0.00 | 0.00 | 0.00 | 0.00 | 0.00 | 0.00 | 0.00 | 0.00 | 0.00 | 0.00 | 0.00 | 0.00 | 0.00 | 0.00 | 0.00 | 0.00 | 0.00 |

| **Figure S4B** | Copies of Luciferase / ng | CAR-T (L) | | | | | | | | | |
| --- | --- | --- | --- | --- | --- | --- | --- | --- | --- | --- | --- |
|  |  | n1 | n2 | n3 | n4 | n5 | n6 | n7 | n8 | Mean | SD |
|  | Day 26 | 0.28 | 0.18 | 0.09 | 0.18 | 0.38 | 0.00 | 0.57 | 0.16 | 0.23 | 0.18 |
|  | Copies of Luciferase / ng | CAR-T (M) | | | | | | | | |  |
|  |  | n1 | n2 | n3 | n4 | n5 | n6 | n7 | Mean | SD |  |
|  | Day 26 | 2.01 | 0.37 | 0.25 | 0.00 | 0.00 | 0.34 | 0.48 | 0.49 | 0.69 |  |
|  | Copies of Luciferase / ng | CAR-T (H) | | | | | | | | |  |
|  |  | n1 | n2 | n3 | n4 | n5 | n6 | n7 | Mean | SD |  |
|  | Day 26 | 0.00 | 0.38 | 0.41 | 0.20 | 0.00 | 0.54 | 0.00 | 0.22 | 0.23 |  |
|  |  |  |  |  |  |  |  |  |  |  |  |
| **Figure S4C** | Copies of CAR / ng | CAR-T (L) | | | | | | | | | |
|  |  | n1 | n2 | n3 | n4 | n5 | n6 | n7 | n8 | Mean | SD |
|  | Day 26 | 5.88 | 4.34 | 10.44 | 6.23 | 9.78 | 6.79 | 6.95 | 48.03 | 12.31 | 14.57 |
|  | Copies of CAR / ng | CAR-T (M) | | | | | | | | |  |
|  |  | n1 | n2 | n3 | n4 | n5 | n6 | n7 | Mean | SD |  |
|  | Day 26 | 21.44 | 41.41 | 32.61 | 5.97 | 9.03 | 28.11 | 5.40 | 20.57 | 14.22 |  |
|  | Copies of CAR / ng | CAR-T (H) | | | | | | | | |  |
|  |  | n1 | n2 | n3 | n4 | n5 | n6 | n7 | Mean | SD |  |
|  | Day 26 | 26.61 | 6.05 | 8.49 | 51.73 | 30.08 | 18.84 | 29.32 | 24.45 | 15.43 |  |

| **Figure S6A** | %PD1^+^ | DLBCL1 | DLBCL2 | CLL1 | CLL2 | CLL3 | HL | Mean | SD |
| --- | --- | --- | --- | --- | --- | --- | --- | --- | --- |
|  | PBMC | 4.49 | 0.91 | 1.21 | 1.89 | 0.16 | 0.91 | 1.60 | 1.52 |
|  | CAR-T | 5.35 | 4.15 | 0.96 | 2.42 | 6.01 | 1.70 | 3.43 | 2.05 |
|  |  |  |  |  |  |  |  |  |  |
|  | %TIM3^+^ | DLBCL1 | DLBCL2 | CLL1 | CLL2 | CLL3 | HL | Mean | SD |
|  | PBMC | 0.04 | 1.77 | 0.04 | 0.02 | 0.34 | 1.77 | 0.66 | 0.87 |
|  | CAR-T | 1.77 | 2.96 | 1.40 | 2.32 | 2.37 | 1.35 | 2.03 | 0.63 |
|  |  |  |  |  |  |  |  |  |  |
|  | %LAG3^+^ | DLBCL1 | DLBCL2 | CLL1 | CLL2 | CLL3 | HL | Mean | SD |
|  | PBMC | 5.05 | 4.89 | 0.50 | 1.56 | 0.88 | 4.89 | 2.96 | 2.20 |
|  | CAR-T | 9.88 | 12.38 | 3.24 | 4.03 | 2.55 | 2.36 | 5.74 | 4.29 |
|  |  |  |  |  |  |  |  |  |  |
|  | %KLRG1^+^ | DLBCL1 | DLBCL2 | CLL1 | CLL2 | CLL3 | HL | Mean | SD |
|  | PBMC | 22.85 | 30.80 | 16.41 | 20.72 | 6.06 | 30.80 | 21.27 | 9.37 |
|  | CAR-T | 11.80 | 14.70 | 2.83 | 4.15 | 6.06 | 4.05 | 7.27 | 4.84 |
|  |  |  |  |  |  |  |  |  |  |
|  | %CD57^+^ | DLBCL1 | DLBCL2 | CLL1 | CLL2 | CLL3 | HL | Mean | SD |
|  | PBMC | 17.03 | 17.20 | 0.88 | 4.55 | 2.52 | 17.20 | 9.90 | 8.02 |
|  | CAR-T | 5.82 | 1.46 | 1.11 | 1.78 | 0.63 | 1.13 | 1.99 | 1.92 |
|  |  |  |  |  |  |  |  |  |  |
| **Figure S6B** | %CD8^+^KLRG1^+^ | DLBCL1 | DLBCL2 | CLL1 | CLL2 | CLL3 | HL | Mean | SD |
|  | PBMC | 67.57 | 72.73 | 35.10 | 48.46 | 45.01 | 46.12 | 52.50 | 14.51 |
|  | CAR-T | 25.90 | 27.35 | 5.49 | 10.44 | 14.94 | 3.43 | 14.59 | 10.15 |

NA: data not available
